# Metal organic frameworks as sorption media for volatile and semi-volatile organic compounds at ambient conditions

**DOI:** 10.1038/srep27813

**Published:** 2016-06-21

**Authors:** Kowsalya Vellingiri, Jan E. Szulejko, Pawan Kumar, Eilhann E. Kwon, Ki-Hyun Kim, Akash Deep, Danil W. Boukhvalov, Richard J. C. Brown

**Affiliations:** 1 Department of Civil and Environmental Engineering, Hanyang University, 222, Wangsimni-Ro, Seoul 04763, Korea; 2 Department of Chemical Engineering, Indian Institute of Technology, Hauz Khas, New Delhi 110 016, India; 3 Department of Environment and Energy, Sejong University, Seoul 05006, Republic of Korea; 4 CSIR-Central Scientific Instrument Organisation (CSIR-CSIO), Chandigarh 160030, India; 5 Department of Chemistry, Hanyang University, 17 Haengdang-dong, Seongdong-gu, Seoul 04763, Korea; 6 Environment Division, National Physical Laboratory, Teddington, Middlesex, TW11 0LW, UK

## Abstract

In this research, we investigated the sorptive behavior of a mixture of 14 volatile and semi-volatile organic compounds (four aromatic hydrocarbons (benzene, toluene, p-xylene, and styrene), six C_2_-C_5_ volatile fatty acids (VFAs), two phenols, and two indoles) against three metal-organic frameworks (MOFs), i.e., MOF-5, Eu-MOF, and MOF-199 at 5 to 10 mPa VOC partial pressures (25 °C). The selected MOFs exhibited the strongest affinity for semi-volatile (polar) VOC molecules (skatole), whereas the weakest affinity toward was volatile (non-polar) VOC molecules (i.e., benzene). Our experimental results were also supported through simulation analysis in which polar molecules were bound most strongly to MOF-199, reflecting the presence of strong interactions of Cu^2+^ with polar VOCs. In addition, the performance of selected MOFs was compared to three well-known commercial sorbents (Tenax TA, Carbopack X, and Carboxen 1000) under the same conditions. The estimated equilibrium adsorption capacity (mg.g^−1^) for the all target VOCs was in the order of; MOF-199 (71.7) >Carboxen-1000 (68.4) >Eu-MOF (27.9) >Carbopack X (24.3) >MOF-5 (12.7) >Tenax TA (10.6). Hopefully, outcome of this study are expected to open a new corridor to expand the practical application of MOFs for the treatment diverse VOC mixtures.

Emissions of volatile organic compounds (VOCs) and semi-volatile organic compounds (SVOCs: volatile fatty acids (VFAs), phenolic, and indolic compounds) and their resulting impact on human health are a major environmental concern^1^,^2^,^3^. Exposure of many of these pollutants has indeed been identified as the causes of diverse (both acute and chronic) health problems^4^. To date, many types of conventional technologies employing diverse classes of materials (e.g., activated carbon, zeolites, and hybrid materials) have been tested for their feasibility toward chemical and physical sorption applications^5^. These technologies have a number of shortcomings or limitations including limited adsorption capacity, toxic end products, and high energy costs for regeneration^6^. In pursuit of a viable means to resolve such problems, the potent role of metal organic frameworks (MOFs) has been recognized due to many advantageous features including high surface area, enhanced gas/vapor adsorption, high catalytic activity, thermal/chemical stability, and tailorable pore sizes. As such, MOFs can be used as excellent adsorbent materials to treat diverse VOCs and VFAs^7^,^8^.

In recent years, the use of pendant and incorporated functional groups (e.g., carboxyl) during post and pre-synthesis has been demonstrated as a simple methodology for increasing adsorption/removal capacity and selectivity toward various gaseous and aqueous phase pollutants^9^,^10^,^11^,^12^,^13^. For example, MOF-199 exhibited good adsorption capacity for benzene as 511 mg g^−1^ at 308 K (1.5 mbar partial pressure and 34% relative humidity (RH))^14^. Likewise, NENU-511 was estimated to have adsorption capacity for benzene as 1.56 g.g^−1^ (or 0.61 g.mL^−1^) at its saturated vapor pressure^15^. The adsorption capacity (mg g^−1^) of toluene and p-xylene on MIL-101 was also measured as 48.8 (at 298 K and 101 kPa)^16^ and 1155 (at 288 K and 5.98 mbar)^17^, respectively. Likewise, some studies were carried out to assess the adsorption behavior of SVOCs on MOFs. The zirconium-based isorecticular MOFs (namely UiO-66 and UiO-66-NH_2_) recorded adsorption of indole at 298 K up to 213 and 312 mg g^−1^, respectively in an aqueous phase based on the strong hydrogen bonding mechanism^18^. The adsorption of phenol on NH_2_-MIL-101(Al) was also measured as 37.6 mg g^−1^ in the aqueous phase at 303 K^19^.

To date, numerous reports have been made to describe the adsorption capacity (and characteristics) of MOFs against common VOCs like aromatic hydrocarbons (e.g., benzene, toluene, and xylene)^14^,^20^,^21^,^22^,^23^,^24^. There is yet a paucity of quantitative data on their capacity toward semi-volatile organic species such as C_2_–C_5_ VFAs, phenolic, and indolic compounds. In fact, the presence of excessive SVOC levels have been recognized as one of the major concerns in: natural gas treatment^25^,^26^,^27^,^28^, animal housing facilities^29^,^30^,^31^, and sewage treatment plants^32^,^33^. Nonetheless, information regarding their treatment approaches is scanty due to their complex physiochemical properties (relative to common VOCs).

In order to fill up our knowledge gaps and to help construct a quality MOF performance database, we investigated the sorption behavior of three well-known MOFs (namely MOF-5, Eu-MOF, and MOF-199) toward a mixture of 14 VOCs (consisting of both volatile and semi-volatile species with varying polarities) at 5 to 10 mPa partial pressures (25 °C). The selection of these three MOFs was made based on their –COOH terminating nature along with easy accessibility (such as preparation methods, storage, and structural characteristics). Because of the commercial availability of these MOFs, our study will help open a route for further investigations for their large-scale applications. As MOFs are also employed as sorbents for analytical purpose (e.g., thermal desorption analysis of gaseous pollutants)^34^,^35^, the performance of these selected MOFs was investigated in reference to three well-known commercial adsorbents (i.e., Tenax TA, Carbopack X, and Carboxen 1000). Hence, our report will also help extend their feasibility as media for gas sampling. In addition, the adsorption mechanism of selected MOFs was also evaluated based on the density functional theory (DFT) and spectroscopic measurements.

## Results and Discussion

### Assessment of conversion efficiency of liquid working standard (LWS) to gaseous working standard (GWS) between different target volatiles

**Figure 1:**
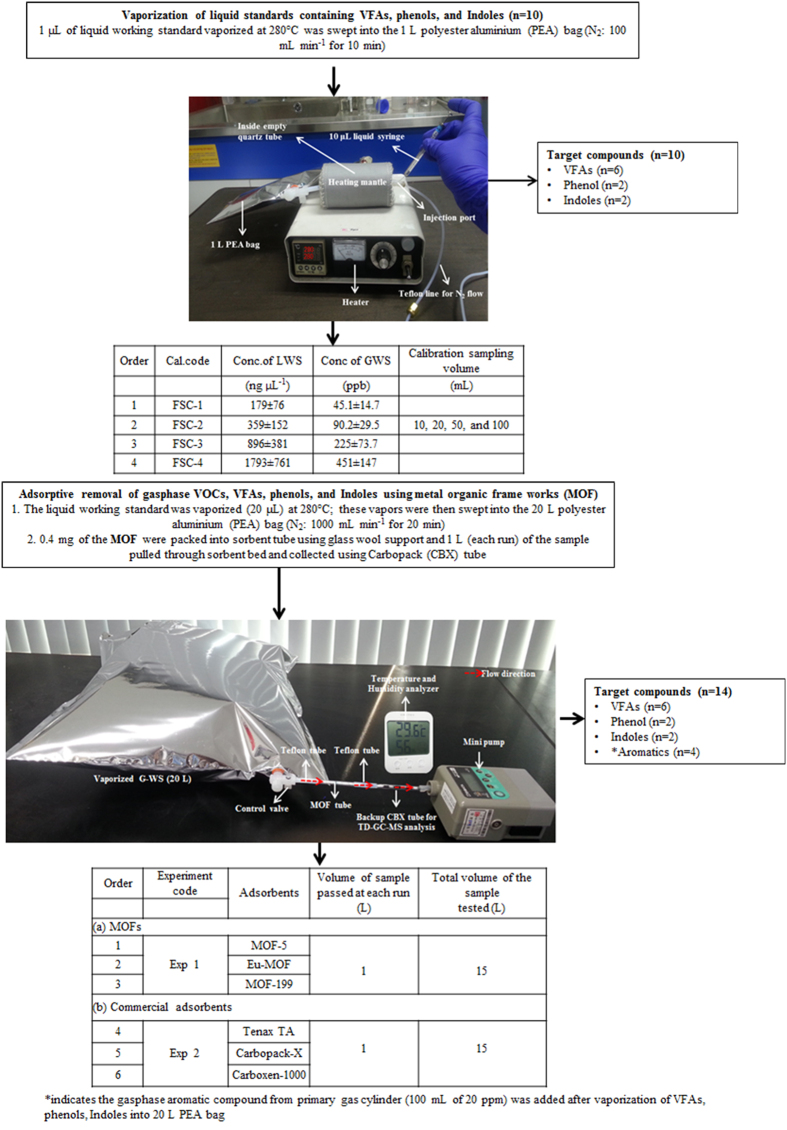
Basic experimental scheme used for the adsorptive removal of volatile and semi-volatile species in this study. (**a**) Pre-experiment for the preparation of gaseous working standards of target VOCs: Conversion from liquids standards and assessment of their conversion efficiency and (**b**) Main experiment: Adsorptive removal of VOCs using three MOFs (Experiment- 1) and three commercial sorbents (Experiment- 2).

In this study, the adsorptive removal of a mixture of 14 VOCs (i.e., four volatile aromatic hydrocarbons (benzene (B), toluene (T), p-xylene (p-X), and styrene (S)), and ten semi-volatile organic compounds (acetic acid (ACA), propionic acid (PPA), isobutyric acid (IBA), butyric acid (BTA), isovaleric acid (IVA), valeric acid (VLA), phenol (PHN), p-cresol (p-C), indole (IN), and skatole (SK)) was assessed using three types of MOFs (MOF-5, Eu-MOF, and MOF-199) at ambient conditions (Tables 1 and 2). Their performance was also compared against three well-known commercial adsorbents, namely: Tenax TA, Carbopack X, and Carboxen-1000. As shown in Fig. 1, three types of experiments were designed and conducted to test the removal efficiency of the MOFs and commercial adsorbents.

In general, the preparation and/or storage of vapor-phase SVOCs (e.g., in the ppb range) are very problematic due to sorptive losses occurring on the surface of materials employed for such purposes (including storage medium, sample tubing, and valves)^36^. Note that the occurrences of such losses depend on the combined effects of several key controlling factors such as volatility (increased losses for semi-volatile species) and storage time. Consequently, primary gas standards for semi-volatile species are generally not available. Thus, the assessment of gas-phase recoveries (prepared by the vaporization of liquid standard) is important due to the complex physiochemical properties of the selected VOCs (Tables S1 and S2)^37^. Table S3 shows the calibration response factor (RF) values (ng^−1^) of the GWSs obtained based on fixed standard concentration (FSC) approaches (FSC-1 to FSC-4)^38^. The RF values of phenolic compounds in the GWS were lower than those of LWS by about 50% (PHN = 6,289 ± 1309 and p-C = 4,694 ± 753 ng^−1^). The maximum loss (about 90%) was observed for indoles (IN = 2,649 ± 573 and SK = 3,137 ± 372 ng^−1^) due to their high adsorption on the polyester aluminum (PEA) bag surface^33^. The relative standard error (RSE (%)) of the VFAs was below 5% in most cases (except ACA), while the phenols and indoles had values below 10%. The rise in RSE (%) for the semi-volatile species is likely due to their physiochemical properties.

The relative recovery (RR) of each target compound in the GWS was calculated by computing the ratio of their RF values between two standard types (Table S4). The VFAs had a mean RR of 52.0 ± 2.01% (n = 6 compounds), while phenols (n = 2) and indoles (n = 2) were 25.0 ± 3.0 and 8.9 ± 0.3%, respectively. This pattern of significant losses is comparable to what was found previously in many studies on various bag types^33^,^36^,^39^,^40^,^41^,^42^. The actual concentrations in each GWS were thus estimated using the RR data (Table S4). For Experiments 1 and 2, 20 L of the GWS in a 20 L PEA bag were used. Tables S5 and S6 provide the RR data for 20 L GWS. The VFAs had a mean recovery of 46.1 ± 2.7% (n = 6 compounds), while those of phenols and indoles (n = 2, 2) were 34.2 ± 16.8 and 18.6 ± 5.3%, respectively.

### Characterization of synthesized MOFs

**Figure 2:**
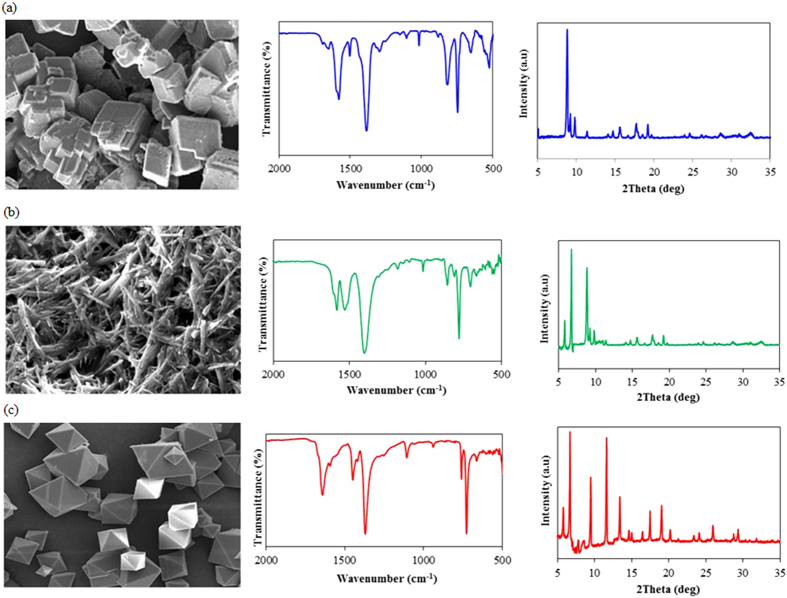
Results of SEM, FTIR, and PXRD analysis of synthesized fresh samples of (**a**) MOF-5, (**b**) MOF-199, and (**c**) Eu-MOF.

The basic properties (e.g., degree of crystallinity, the chemical functionality, the morphology, and the surface area) were examined using powder X-ray diffraction studies (PXRD), Fourier transform infrared spectroscopy (FTIR), scanning electron microscopy (SEM), and N_2_ adsorption and desorption studies, respectively (Fig. 2). The two MOF-5 FTIR peaks at 1385 and 1579 cm^−1^ indicated the attachment of the carboxylate ligand to the Zn_4_O center^43^. Likewise, the peaks located near 816, 746, and 653 cm^−1^ confirmed Zn-O stretching modes. IR bands at 1502, 1294, and 653 cm^−1^ indicated a random dimethyl formamide (DMF) distribution in the MOF-5 framework structure^44^. The band located at 3607 cm^−1^ indicated adsorbed water molecules. The Eu-MOF showed peaks at 1530 and 1582 cm^−1^, which were attributed to the symmetric and asymmetric C = O stretching vibrations, respectively. Compared with the free carboxyl groups (~1700 cm^−1^), the presence of a low wavenumber peak indicates the possible coordination between carboxyl groups of the ligand with Eu^5+ ^ (Ref. 45). The strong peaks around 665 and 779 cm^−1^ were attributed to the Eu-O stretching vibrations resulting from successful coordination between Eu^3+^ and the carboxylic group^46^. The FTIR spectra of the synthesized MOF-199 showed a narrow band around 2931 cm^−1^ indicating the presence of –CH_3_ groups in DMF. The peaks at 760.2 and 727.9 cm^−1^ were due to Cu coordination to BTC, while those located at 1106.9 and 939.7 cm^−1^ were due to C-O-Cu stretching vibrations^47^. The asymmetric stretching vibrations of the carboxylate group were observed at 1641.9 cm^−1^, whereas symmetric modes were observed at 1442.3 and 1372.9 cm^−1 ^48.

The observed PXRD peaks of Eu-MOF were in good agreement with the reported data^46^. The main MOF-199 diffraction peaks (at 2θ = 6.7, 9.4, 11.6, 13.4, 17.4, 19.0, and 29.4) confirmed its structure^49^,^50^. Likewise, the MOF-5 peaks are also in excellent agreement with the literature using a similar synthesis procedure^3^. As seen in Fig. 2, SEM images of MOF-5 showed cubic crystals^3^, while MOF-199 crystals have an octahedral morphology in agreement with Zhao *et al*.^14^. In contrast, Eu-MOF exhibited a grass-like structure (~20 μm) in agreement with its reported morphologies^46^.

The Langmuir surface area of the synthesized MOF-5 (535 m^2^ g^−1^ ) was lower than that reported previously (895 m^2^ g^−1^)^3^; this could be due to solvent molecules being trapped in the MOF-5 cages^3^ with the estimated total pore volume of 0.22 cm^3^ g^−1^. In the case of Eu-MOF and MOF-199, the Langmuir surface area values were 281 and 1591 m^2^ g^−1^, respectively, while the corresponding total pore volume was 0.95 and 0.46 cm^3^ g^−1^, respectively; these results are also in good agreement with previous work^51^,^52^. The corresponding N_2_ adsorption-desorption isotherms are shown in Fig. S1.

### Adsorption capacity

**Figure 3:**
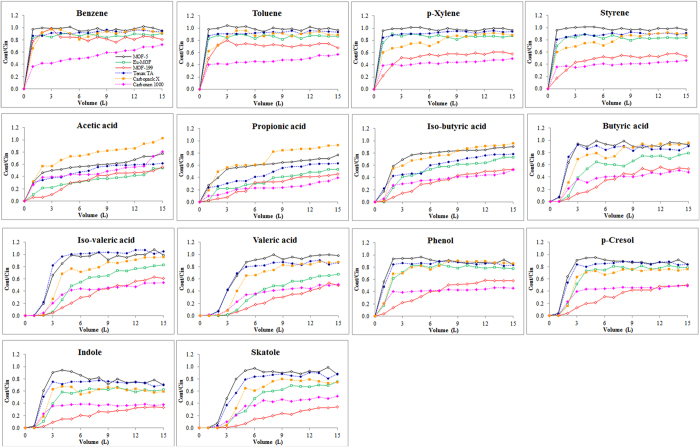
Breakthrough curves of different MOFs (MOF-5, Eu-MOF, and MOF-199) with respect to selected reference materials (Tenax TA, Carbopack-X, and Carboxen-1000) for removing 14 gaseous pollutants. Note the use of the same legend as for benzene panel for all the six sorbents.

The equilibrium adsorption capacities (q) were calculated according to


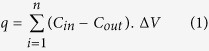


where q (mg g^−1^) is the adsorption capacity, C_in_ and C_out_ are inlet and outlet concentrations of gaseous compounds, respectively, ΔV is sample volume (1 L) (Table 3). It should be noted that sorbent saturation for some tested MOFs was not attained within 15 L sample loading, e.g., skatole on MOF-199 (Fig. 3). To allow comparative analysis of performance between MOFs and commercial sorbents, breakthrough was also determined. The estimated equilibrium adsorption capacities of MOFs are only applicable to partial pressures of ~0.01 Pa; it should thus be noted that such estimates are unlikely to directly correspond to the maximum MOF adsorption capacities measured at higher VOC partial pressures (e.g., 10,000 Pa).

Although quartz wool (QW) is used to support and hold the sorbent media in the sorbent bed, it can also act as a very weak sorbent^53^. Moreover, in many studies carried out with sorbent tube sampler, QW is generally used as a supporting medium for analytical purposes. Their affinity is minimal for aromatic hydrocarbons but found to be maximum for SVOCs and PAHs^54^. Hence, in this study we tried to evaluate the sorptive losses of SVOCs due to the quartz wool by measuring its sorptive capacity like other sorbent materials.

The results showed contrasting patterns between different compounds for both empty and QW filled sorbent beds. Analyte loss was negligible or the smallest for aromatic hydrocarbons, while it was rather moderate to large for other targets like VFAs, phenolics and indolic species. For ACA, the detected mass (m_D_) exiting the sorbent bed packed with ~50 mg QW can be expressed as: slope (0.398)*(mass loaded (m_L_) + intercept). The intercept of −12.8 ng corresponds to a detection threshold (DT) of 32 ng^55^. DT is the minimum mass loaded at which breakthrough (BT) can occur. DT ranged from ~0 (aromatic hydrocarbons) to ~400 ng (p-cresol). As such, the slopes calculated for m_D_ vs. m_L_ (if m_L_ > DT) varied from 0.398 (ACA) to ~1 (aromatic hydrocarbons). As such, it is important to employ an exact analytical method to estimate the errors associated with the sampling devices and the sorbent medium when measuring ambient indoor air quality. Hence, to adjust the effect of all unknown factors contributing to the analyte losses (silicone rubber and other components), the loss fraction (LF) of analyte mass needs to be subtracted from the total mass sorbed (*q*_*tot*_) to obtain the mass of each VOC sorbed (*q*_s_) only on a tested sorbent (Eqn. 2),


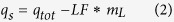


As there was some variability in assembling the apparatus, the *LF* will vary from experiment to experiment. The determination of the *LF* was done graphically as shown in Fig. S2 for three reference sorbents (Tenax TA, Eu-MOF, and Carbopack X). *LF* values were adjusted to obtain near horizontal plots for both q_s_ and the Henry’s law constant (HLC, mol kg^.−1^ Pa^−1^) (Fig. S2); e.g., valeric acid/Carbopack X (Fig. S2 (a)). Here,


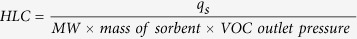


where MW is the molecular weight of a given VOC (g mol^−1^). The detection threshold was not considered in Eqn. (2), and it was set to 0; the obtained HLC adsorption, capacity, and LF data are summarized in Table S7. The *LF* values varied from 0.05 (benzene) to 0.62 (indole).

The mean equilibrium adsorption capacities (after QW correction) for the four aromatic (C_6_–C_8_) hydrocarbons were noticeably lower (MOF-199: >13.8 and Eu-MOF: 3.56 mg g^−1^) than those of the VFAs (MOF-199: 18.6 and Eu-MOF: 15.3 mg g^−1^). However, MOF-199 demonstrated maximum equilibrium adsorption capacities for phenols (27.9 mg g^−1^) and indoles (12.2 mg g^−1^) (Table 3). If expressed as Henry’s constants (mol kg^−1^ Pa^−1^) (Table S7), the MOF-199 values for benzene, toluene, and p-xylene in the present work (at 298 K) are comparable to those of MOF-1, MIL-47, and IRMOF-1 extrapolated to 298 from 448 K using the van’t Hoff equation and reported isosteric ΔH_ab_^56^. For instance, in the case of toluene, the Henry’s constant of MOF-199 (>5.3), in this work is comparable to that of MOF-1 (14.2)^56^. However, for the benzene/MOF-199 system at 298 K, our Henry’s constant (>2.1) at a benzene pressure of 0.0081 Pa is significantly larger than the 5.08 × 10^−2^ (at a benzene pressure of 44 Pa) calculated from the data given by Yang *et al*.^57^. Qualitatively, our Henry’s constants for the aromatic hydrocarbons are in fair agreement with the literature, after appropriate adjustment for low partial pressures^58^.

Although open electron deficient metal sites are reactive species in MOFs, some of the electron rich species (i.e., Lewis bases like benzene) had very low affinity. Indeed, the square 4-coordinated open copper metal sites may be responsible for the high adsorption of MOF-199. These sites may also act as a Lewis acid/base depending on the functionality of the framework. This mechanism illustrates the heterogeneity and guest size dependency (size selectivity effect) of MOF-199 toward the target compounds. In addition, the adsorption mechanism of the Eu-MOF can be accounted for by defect-free nano sized (~2 nm) coordination sites in the framework and a comparatively low adsorption energy of the partially filled 4f electrons during adsorption^51^.

The MOF-5 exhibited remarkably reduced equilibrium capture capacities for all target analytes (Table S7) (aromatic hydrocarbons: ~0 mg.g^−1^, VFAs: 7.33 mg.g^−1^, phenols: >1.50 mg.g^−1^, and indoles: 3.90 mg.g^−1^). Consequently, the measured concentrations in the outlet were in some occasions higher than those of the inlet (C_out_/C_in_ = ~1.2) (Fig. 3). This is because in a multicomponent analysis, compounds with a weaker MOF interaction can be challenged and displaced by a more strongly adsorbing species during dynamic adsorption. This phenomenon was evident for aromatic compounds (B, T, and p-X) and some VFAs (IVA and VLA). We suspected that this observation indicates the occurrence of “roll-up”. This poor performance of MOF-5 may be attributable to the highly cubic pore structure, which is reported to diminish the framework functionality during dynamic adsorption^7^.

To the best of our knowledge, there have been virtually no efforts to address the relative adsorption behavior of MOFs for different components with various volatilities as used in this study. Hence, to establish some comparative reference data, the performances of three well-known commercial adsorbents were also analyzed. Among those, Carboxen-1000 showed the highest mean equilibrium adsorption capacity for all target VOCs (aromatics: >22.2 mg g^−1^, VFAs: >23.4 mg g^−1^, phenols: >20.0 mg g^−1^, and indoles: 2.80 mg g^−1^). In contrast, such values were considerably reduced in the case of Carbopack X (aromatics: 3.74 mg g^−1^, VFAs: 10.2 mg g^−1^, phenols: 5.20 mg g^−1^, and indoles: 4.10 mg g^−1^) and Tenax TA (aromatic hydrocarbons: 0.96 mg g^−1^, VFAs: 3.85 mg g^−1^, phenols: 2.50 mg g^−1^, and indoles: 3.30 mg g^−1^). The increased equilibrium sorption capacity of the Carboxen-1000 may be due to its high surface area (1200 m^2^ g^−1^) compared to the other two commercial adsorbents (e.g., Tenax TA (36 m^2^ g^−1^) and Carbopack X (240 m^2^ g^−1^)).

### Estimation based on DFT calculations

**Figure 4:**
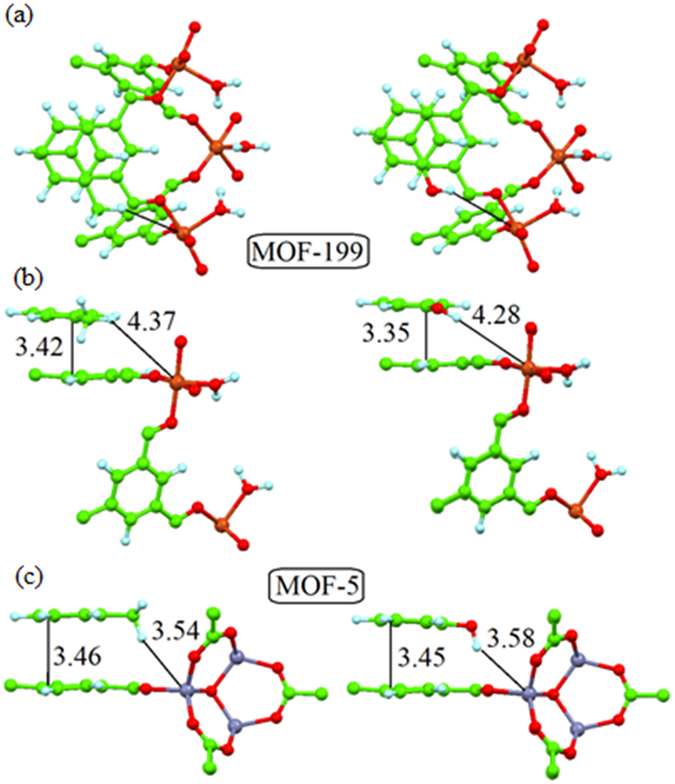
Atomic structure of MOF before and after adsorption of VOCs: Top (**a**) and side (**b,c**) views of optimized atomic structures of MOF-199 (**a,b**) and MOF-5 (**c**) with adsorbed toluene (left) and phenol (right) molecules. Interatomic distances indicated on the figure are in Å. Parts of the atomic structure of both MOFs are omitted for clarity.

The interactions between MOFs and target compounds can be evaluated more meaningfully by considering the structure of the target species. For instance, toluene, phenol, and indole contain six-member aromatic rings. However, phenol (and butyric acid) has a polar –OH group, while indole has a polar N-H group in a five-member ring. Results of DFT calculations (Fig. 4a–c) demonstrate a number of bonding modes for the same compound with different MOFs. In the case of MOF-5 (with small pores), its interaction with the six-membered ring compounds is similar to AA stacking in graphite when both rings were placed one above the other. In contrast, the less dense MOF-199 showed more energetically favorable coupling (similar to the AB-stacking in graphite) when the rings of both phenol and toluene were shifted by the host MOF rings (Fig. 4a,b). This stacking information allowed us to assess different energies of π-π interactions between molecules and the benzene-ring structure of MOF-199. In addition, it may also be ascribable to the favorable interactions between the polar parts of the guest molecules and the Cu-ions of the MOF-199. In the case of MOF-5, no such interactions between the polar parts of the guest molecules and the Zn-ions were clearly seen based on the orientation of -OH groups (Fig. 4c). This contrasts to that of MOF-199 in which interactions occur despite the larger H-Cu distance orientation of -OH groups.

We are able to explain the differences in the calculated change in Gibb’s free energy (ΔG_ad_) values between MOF-5 and MOF-199 (Table S8) based on the observed differences in adsorption. The polarity of the guest molecule influenced the MOF-5 ΔG_ad,_; i.e., toluene was −4.0 kJ mol^−1^ while phenol was −10.2 kJ mol^−1^. This result suggests that the replacement of a nonpolar –CH_3_ group with a polar –OH can enhance the adsorption efficiency. This is even more effective for MOF-199 due to the presence of an additional bond between the guest molecule and Cu-ion, while the influence of polarity almost doubled the binding energies (Table S8) (e.g., ΔG_ad_ value for toluene was −39.9 kJ mol^−1^, while those of phenols and indoles were −53.9 and 56.8 kJ mol^−1^, respectively). Our estimated 298 K ΔG_ad_ value for toluene/MOF-199 of −39.9 kJ mol^−1^ is comparable to 298 K ΔG_ad_ values for MOF-1, MOF-47, and IRMOF of −46.1, −44.2, and −25.3 kJ mol^−1^, respectively^56^. To this end, the selection of the MOFs for the adsorption process can be highly dependent on the availability of the π-orbitals for guest interaction as well as favorable host-guest interactions based on the framework polarity.

The microscopic mechanism of the host-guest interaction was also investigated in terms of changes in the chemical structural properties before and after the adsorption of guest molecules on MOFs by infrared spectroscopy (Fig. S3). The framework structure of all three MOFs remained intact before and after adsorption. In addition, the FTIR results suggest favorable π-π interactions of the guest molecules with COO^−^ and C = C regions in the MOFs. The detailed description was given in the supplementary information.

## Conclusions

The adsorption capacities of three MOFs (MOF-5, Eu-MOF, and MOF-199) toward a mixture of 14 gaseous volatile and semi-volatile organic species (partial pressures ~0.01 Pa) were analyzed and compared against three conventional sorbents (namely Tenax TA, Carbopack-X, and Carboxen-1000) at ambient conditions. In conclusion, MOF-199 showed the highest equilibrium adsorption capacity for all 14 VOCs of ~72 mg.g^−1^ followed by Eu-MOF (28 mg.g^−1^) and MOF-5 (13 mg.g^−1^) when 15 L of a ~100 ppb (~0.01 Pa) gaseous standard was loaded at ~25 °C. Our experimental results suggest that adsorption mechanism for MOF-199 was most favorable due to the presence of open metal sites in the framework. In addition, our DFT simulation analysis showed that presence of strong π-π interactions (MOF-199 >Eu-MOF (27.9) >MOF-5) and polarity of the guest molecule were the key factors influencing the sorption. Furthermore, the irreversibility of the MOF adsorption needs to be validated further (despite the fact that the adsorption Gibbs free energies provide an indication) and should be compared to conventional sorbents such as Carboxen-1000 and Tenax-TA. Most importantly, our study results are expected to provide valuable insights into the sorptive capacity of MOFs against many important hazardous and/or odorous volatile pollutants in indoor environment.

## Materials and Methods

### Experimental design

The synthesis of MOF-5 followed previous work with some modifications^3^. Likewise, nano-sized Eu-MOF was synthesized according to the procedure of Choi *et al*.^51^, while the blue uniform crystals of the MOF-199 were synthesized by following the procedure of Millward *et al*.^52^.

The synthesized MOF products were characterized and verified by using PXRD (Bruker D8 Focus diffractometer), SEM (Mini-SEMSNE-4000M), and Attenuated reflectance method (ATR)-FTIR spectroscopy (PerkinElmer L1600400–IR spectrometer). A diffraction analysis was performed on a standard glass slide for background correction. The data were recorded over a 2θ range of 5–35°. Specific surface area and pore parameters were estimated from N_2_ adsorption/desorption isotherms at 77 K using a Micrometrics ASAP 2010. Prior to experiments, MOF samples were heated at 423 K for 8 h under a reduced pressure of 133.3 Pa to remove adsorbed water and gases. A detailed description of the synthesis of the MOFs is provided in the supporting information.

### Relative recovery from bags

As a part of the pre-experiment, we assessed the reliability of GWS containing the 10 semi-volatile target analytes (including VFAs (n = 6), phenols (n = 2), and indoles (n = 2)) by comparing changes in their sensitivity, i.e., their calibration response factors (RF) (ng^−1^) derived from both liquid (LWS) and gaseous (GWS) standards (Fig. 1A). The gas phase recoveries of LWS were calculated as follows:


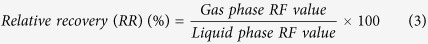


where relative recovery from bags, RF is the response factor (ng^−1^). Detailed experimental procedures and derived RR analysis results are summarized in the supplementary information in Tables S1 to S6. BT experiments were performed to assess the competitive adsorption capacity of the three synthesized MOFs for the mixture of 14 target analytes (Fig. 1B).

Prior to the breakthrough experiments, the MOFs were treated at 100 °C for 1 h under a nitrogen purge at 100 mL min^−1^ to remove the possible contaminants. A gaseous mixture containing low-ppb target compounds at 1 atm was pulled through the sorbent bed (89 mm length, 4 mm id, and 6 mm in od) packed with 0.4 mg of a given MOF using a purge flow rate of 200 mL min^−1^. The inlet concentration of the VOC (C_in_) was ~100 ppb (Table S9). The effluent gas stream was sampled using a three-bed (Carbopack C, B, and X) sorbent tube (ST) at 5 min intervals for ST/thermal desorption/gas chromatography/mass spectrometric (ST/TD/GC/MS) analysis to determine the outlet VOC concentration (C_out_).

### Comparison of sorptive capacity between MOFs and commercial adsorbents

To date, there is a dearth of data on MOF sorption performance for various gaseous species, especially SVOCs. To learn more about the adsorption behavior of VOCs on MOF, we conducted a series of comparative sorptive removal experiments using three MOFs (Experiment 1) and three well known commercial adsorbents as a reference (Experiment 2, Fig. 1B). The mass of all six sorbents used in the experiments was kept constant (0.4 mg) to facilitate comparisons of breakthrough concentrations, breakthrough volumes, and equilibrium adsorption capacity. Detailed descriptions of the experimental setup (Experiment 1 and 2) are provided in the supplementary information.

### Density functional theory (DFT)

The interactions between the MOF and the guest molecules were studied using DFT calculations. The pseudo-potential code SIESTA^59^ model was applied for this purpose. All calculations were conducted using the generalized gradient approximation (GGA-PBE) with spin-polarization^60^ by taking into account the +*vdw* correction^61^ which is required for describing weak interactions. Full optimization of atomic positions was performed. During the optimization, the ion cores are described by norm-conserving non-relativistic pseudo-potentials^62^ with cut-off radii of 2.43, 2.25, 1.15, 1.14, 1.45, and 1.25 au for Zn, Cu, O, C, N and H, respectively. The wave functions were expanded with a double-ζ plus polarization basis of localized orbitals for all species excluding hydrogen. Optimization of the force and total energy was performed with an accuracy of 0.04 eV/Å and 1 meV, respectively. All calculations were carried out with an energy mesh cut-off of 300 Ry and a k-point mesh of 4 × 4 × 4 in the Mokhorst-Park scheme^63^. The calculation was conducted for realistic atomic structures of MOF-5 and MOF-199 taken from the Cambridge Database (CCDC). The DFT-based 0 K change in adsorption enthalpy (ΔH_ad_) was calculated by


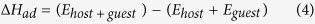


Here, E_host+guest_ is the total energy of MOF with adsorbed guest molecules, E_host_ is the total energy of the pure MOF, and E_guest_ is the energy of the guest molecule in the gas-phase. The adsorption Gibbs free energy (ΔG_ad_ at 298 K) was calculated using the 0 K ΔH_ad_ (with no thermal and zero-point energy corrections) and the estimated adsorption entropy change (ΔS_ad_) by following the empirical procedure of Campbell and Sellers^64^,


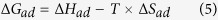


## Additional Information

**How to cite this article**: Vellingiri, K. *et al*. Metal organic frameworks as sorption media for volatile and semi-volatile organic compounds at ambient conditions. *Sci. Rep.* **6**, 27813; doi: 10.1038/srep27813 (2016).

## Supplementary Material

Supplementary Information

## Figures and Tables

**Table 1:** List of target compounds selected for the evaluation of adsorptive removal by MOFs (MOF-5, Eu-MOF, and MOF-199) and reference mesoporous (Tenax TA, Carbopack-X, Carboxen-1000) materials.

Order	Group	Compounds	Short name	MW (g mol^−1^)	Density (g cm^−3^)	Boiling point °C	Formula	CAS number	Extracted ion chromatogram^a^	Odor Threshold (ppm)^b^
1	Aromatic hydrocarbons	Benzene	B	78.1	0.877	80	C_6_H_6_	71-43-2	78	2.70
2		Toluene	T	92.1	0.870	111	C_7_H_8_	108-88-3	91, 92	0.33
3		p-Xylene	p-X	106	0.861	138	C_8_H_10_	106-42-3	91, 106	0.058
4		Styrene	S	104	0.909	145	C_8_H_8_	100-42-5	91, 106	0.035
5	Carboxyl	Acetic acid	ACA	60.1	1.049	118	C_2_H_4_O_2_	64-19-7	43, 45, 60	6.00
6		Propionic Acid	PPA	74.1	0.990	141	C_3_H_6_O_2_	79-09-04	73, 74	5.70
7		i-Butyric Acid	IBA	88.1	0.970	154	C_4_H_8_O_2_	79-31-2	73	0.19
8		n-Butyric Acid	BTA	88.1	0.960	164	C_4_H_8_O_2_	107-92-6	60	1.50
9		i-Valeric Acid	IVA	102	0.930	177	C_5_H_10_O_2_	503-74-2	60	0.078
10		n-Valeric Acid	VLA	102	0.940	186	C_5_H_10_O_2_	109-52-4	60	0.037
11	Phenols	Phenol	PHN	94.1	1.070	182	C_6_H_6_O	108-95-2	94	5.60
12		p-Cresol	p-C	108	1.035	202	C_7_H_8_O	106-44-5	107, 108	0.056
13	Indoles	Indole	ID	117	1.175	254	C_8_H_7_N	120-72-9	117	0.30
14		Skatole	SK	131	1.100	266	C_9_H_9_N	83-34-1	130, 131	0.006

^a^m/z values used for quantification (selected ion monitoring).

^b^Refers to^65^.

**Table 2:** Fundamental parameters describing the adsorbent used for adsorption experiments (Experiments 1 and 2).

Order	Adsorbent name	Adsorbent class	Source	Langmuir surface area (m^2^ g^−1^)	Total pore volume cm^3^ g^−1^
1	MOF-5	Crystalline porous materials	In house laboratory synthesis	535	0.22
2	Eu-MOF	Crystalline porous materials	In house laboratory synthesis	281	0.95
3	MOF-199	Crystalline porous material	In house laboratory synthesis	1591	0.46
**(*2*)*****Group 2: Commercial adsorbents***^a^					
4	Tenax TA	Porous polymer	Supelco, USA	~35^b^	–
5	Carbopack X	Graphitized carbon	Supelco, USA	~240	–
6	Carboxen-1000	Carbon molecular sieves	Supelco, USA	~1200	–

^a^Indicates commercial adsorbents purchased from Sigma-Aldrich.

^b^The presented surface area corresponds to BET surface area provided by the vendor (https://www.sigmaaldrich.com/content/dam/sigma-aldrich/docs/Supelco/General_Information/t402025.pdf

**Table 3:** Equilibrium adsorption capacities of different MOFs and commercial adsorbents (after correction for quartz wool uptake) at given partial pressure.

Equilibrium adsorption capacities (mg g^−1^)								
Order	Compounds	Partial pressure (mPa)	MOFs			Commercial adsorbents^a^		
			MOF-5	Eu-MOF	MOF-199	Tenax TA	Carbopack-X	Carboxen-1000
**A. Aromatic HCs**								
1	B	8.6	-b	1.00	>1.1^c^	0.25	0.42	4.80
2	T	6.8	-	0.95	>2.6	0.24	0.62	5.40
3	p-X	6.0	-	0.76	>5.2	0.27	1.50	>6.0
4	S	5.5	-	0.85	>4.9	0.20	1.20	>6.0
	**Sum**		**-**	**3.56**	**>13.8**	**0.96**	**3.74**	**>22.2**
**B. VFAs**								
5	ACA	5.3	0.51	0.83	0.89	-	0.20	-
6	PPA	5.4	0.78	1.10	2.10	-	0.33	4.00
7	IBA	6.2	0.74	1.40	2.40	0.20	1.00	3.40
8	BTA	6.6	1.00	3.00	3.10	0.65	2.30	4.50
9	IVA	5.9	1.90	3.90	4.10	1.30	3.20	>5.5
10	VLA	5.2	2.40	5.10	6.00	1.70	3.20	>6.0
	**Sum**		**7.33**	**15.3**	**18.6**	**3.85**	**10.2**	**>20.0**
**C. Phenols**								
11	PHN	12	-	1.50	13.0	0.70	2.50	>10.0
12	p-C	9.8	1.50	3.00	15.0	1.80	2.70	>10.0
	**Sum**		**1.50**	**4.50**	**28.0**	**2.50**	**5.20**	**>20.0**
**D. Indoles**								
13	IN	5.0	1.40	1.50	4.50	1.10	1.80	0.70
14	SK	4.8	2.50	3.00	7.70	2.20	3.30	2.10
	**Sum**		**3.90**	**4.50**	**12.2**	**3.30**	**5.10**	**2.80**
**15**	**Total**		**12.7**	**27.9**	**>71.7**	**10.6**	**24.3**	**>68.4**

^a^Adsorption capacities based on reported BTVs (http://www.sisweb.com/) at a partial pressure of 10 mPa.

^b^Adsorption capacities were not measurable.

^c^Saturation for those compounds were not attained within 15 L sample loading.
