# Supplementary material for: Metal organic frameworks as sorption media for volatile and semi-volatile organic compounds at ambient conditions
Source: Sci Rep. 2016 Jun 21;6:27813. doi: 10.1038/srep27813 (PMC4914961; doi:10.1038/srep27813)
Supplement: Supplementary Information [file srep27813-s1.doc]

**Supporting Information**

**Metal organic frameworks as sorption media for volatile and semi-volatile organic compounds at ambient conditions**

Kowsalya Vellingiri^1^, Jan E. Szulejko^1^, Pawan Kumar^2^, Eilhann E. Kwon^3^, Ki-Hyun Kim^1,*^,

Akash Deep^4^, Danil W. Boukhvalov^5^, Richard J. C. Brown^6^

^1^Department of Civil and Environmental Engineering, Hanyang University, 222, Wangsimni-Ro, Seoul 04763, Korea; ^2^Department of Chemical Engineering, Indian Institute of Technology, Hauz Khas, New Delhi 110 016, India; ^3^Department of Environment and Energy, Sejong University, Seoul 05006, Republic of Korea; ^4^CSIR-Central Scientific Instrument Organisation (CSIR-CSIO), Chandigarh 160030, India; ^5^Department of Chemistry, Hanyang University, 17 Haengdang-dong, Seongdong-gu, Seoul 04763, Korea; ^6^Environment Division, National Physical Laboratory, Teddington, Middlesex, TW11 0LW, UK.

**1 Methods and materials**

**Experimental design**

To analyze the conversion efficiency between liquid and gaseous standard phases (for VFAs, phenolic, and indolic compounds) the relative recovery was calculated (pre-experiment). To this end, the inlet of the polyester aluminum (PEA) bag containing prepared gaseous working standard (GWS) was connected to the sorbent tube (ST) (ID (4mm), and the total sorbent bed length (89 mm) was packed with 70, 50, and 50 mg of Carbopack C, B, and X, respectively. Quartz wool (QW) was used as partition medium between each sorbent, and the outlet was connected to a vacuum pump (Sibata, ∑MP-30, Japan). Flow was controlled using a mass flow controller (MFC). The sampling flow rate was fixed at 100 mL min^-1^, and the sample loading volumes for all four concentrations of GWS used (1A to 1D) were 10, 20, 50, and 100 mL.

A ~100 ppb mixture of GWS was prepared in a 20 L PEA bag for the adsorption analysis. The 20 L PEA bag was connected via Teflon tubing to the ST packed with 0.4 mg of MOF embedded in QW in a quartz tube (ID: 4 mm, OD: 6 mm, and length: 89 mm) (Experiment 1). The PEA bag contents were pulled through the ST using a mini-pump. The flow rate was fixed at 200 mL min^-1^ for 5 min. In each run, 1 L of the GWS was pulled through the MOF/ST and ST over a total of 15 runs (15 L). A separate ST was used for each run. The initial and final concentration of the GWS in the PEA bag was analyzed in the same manner before and after the adsorption experiment. Each experiment was conducted quickly (in less than 100 min) to minimize sorptive losses in the PEA bag. The three commercial adsorbents (0.4 mg each) were embedded in QW and tested in the same manner as in Experiment 1.

Calibration and the quality assurance (QA) data were obtained from an analysis of the LWS. In order to analyze the LWS, the inlet of the ST was connected via Teflon tubing to a 1 L PEA bag filled with ultrapure nitrogen (>99.999%). The outlet of the ST was then connected to a Sibata mini vacuum pump (∑MP-30, Japan). Subsequently, 1 μL of the final L-WS was injected onto the ST using a 5 μL liquid syringe (SGE Analytical science, Australia) via a temporary injection port connecting the inlet of the ST with a PEA bag. Back-up gas (from the PEA bag) was simultaneously delivered to the ST (flow rate = 100 mL min^-1^ for 3 min). To maintain the consistency throughout the experimental stages, the three-bed ST was prepared in the same manner each time by packing Carbopack-C (70 mg), – B (50 mg), and –X (50 mg) (Supelco, USA) into empty quartz tubes. A total of 22 ST were identically prepared and used throughout the experiment.

**Preparation of liquid and gaseous working standards**

To measure the adsorptive removal capacity of the three MOFs selected in this work, a total of 14 analytes were selected as target compounds in this study (**Table S1**). These analytes included: (1) six VFAs: acetic acid (ACA), propionic acid (PPA), i-butyric acid (IBA), n-butyric acid (BTA), i-valeric acid (IVA), and n-valeric acid (VLA), (2) two phenolic compounds: phenol (PHN) and p-Cresol (p-C), (3) two indolic compounds: indole (IN) and skatole (SK), and (4) four VOCs: benzene (B), toluene (T), p-xylene (p-X), and styrene (S).

**Preparation of liquid standards**

Reagent grade chemicals (RGCs) of all 14 target compounds (98.0-99.9%) were purchased from Sigma-Aldrich, USA and methanol was purchased from Burdick & Jackson, USA. The primary standards (PS) were prepared separately as PS-1, PS-2, and PS-3 by mixing RGCs with methanol **(Table S1A)**: (1) PS-1 was for phenols and indoles (n=4), (2) PS-2 was for VFAs (n=6), and (3) PS-3 was for aromatics (n=4). As the PS-1 compounds are solid phase chemicals, they were prepared by dissolving the solids in methanol to make a 20 mL solution in a volumetric flask. The PS-2 chemicals are semi-volatile organic compounds and 1 mL of each compound was mixed with methanol to make a 20 mL solution. The PS-3 chemicals are highly volatile organic compounds; each 500 μL of the PS-3 chemicals was also mixed with methanol to make a 20 mL solution. The mean concentrations of PS-1, PS-2 and PS-3 were 21.8 ± 0.50, 48.2 ± 2.12, and 26.8 ± 0.14 μg μL^-1^, respectively. For the pre-experiment, the first liquid working standard (1^st^ L-WS) was made by mixing 250 μL of each PS-1 and PS-2 with methanol (8750 μL) (mean ± SD concentration): (1) VFAs = 1204 ± 53 ng μL^-1^, (2) phenols and indoles = 2679 ± 0.95 ng μL^-1^, and 2673 ± 19.2 ng μL^-1^, respectively **(Table S1 B)**. The second liquid working standard (2^nd^ L-WS) of all targets used for conversion into the GWS was prepared by diluting the 1^st^ L-WS with methanol to yield four different concentrations with mean values ranging from 120 ± 53.0 to 1204 ± 53.0 ng μL^-1^ (VFAs) and from 268 ± 1.17 to 2676 ± 11.7 ng μL^-1^ (phenols and indoles). The final L-WS for calibrations were prepared by diluting the second L-WS (mean ± SD concentration (ng μL^-1^): (1) VFAs: 1.20 ± 0.05 to 179 ± 76.1 and (2) phenols and indoles: 2.68 ± 0.01 to 268 ± 1.17). The resulting eight point calibration relationship was used to calculate the relative recovery (RR) of the gaseous standards containing VFAs, phenols, and indoles.

For Experiment 1, the 1^st^ L-WS of VFAs, phenols, and indoles were prepared a manner similar to the pre-experiment. In Experiment 1, four aromatic compounds were also included to study the sorptive removal properties. For this, PS-3 with 5000 μL of the BTXS mixture was added into methanol to make a 20 mL solution. The 2^nd^ L-WS of VFAs, phenols, and indoles (used for vaporization) was prepared by mixing 5000 μL of the 1^st^ L-WS with methanol to make a 20 mL solution (896 ± 380 ng μL^-1^). The 2^nd^ L-WS of the aromatic compounds was prepared by diluting 150 μL of the 1^st^ L-WS into 9850 mL of methanol (327 ± 7.51 ng μL^-1^) **(Table S1 C)**. The final L-WS for calibration was prepared by diluting the 2^nd^ L-WS with methanol to yield a concentration range of (1) VFAs: 6.02 ± 0.27 to 361 ± 15.9 ng μL^-1^, (2) phenols and indoles: 13.4 ± 0.06 to 803 ± 3.50 ng μL^-1^, and (3) aromatics: 3.27 ± 0.07 to 327 ± 7.50 ng μL^-1^. The eight-point calibration relationship and response factors (RF) obtained were used for Experiment 1 and 2.

**Preparation of gaseous-phase working standard**

For pre-experiment 1, the G-WS was prepared by vaporizing 1 μL of the 2^nd^ L-WS (120 ± 53.0 to 1204 ± 53.0 ng μL^-1^ for VFAs and 268 ± 1.17 to 2676 ± 11.7 ng μL^-1^ for phenols and indoles). This was collected into a 1 L PEA bag. The inlet and outlet of the empty quartz tube were connected to a gas cylinder (via a fine, low dead volume valve) containing ultra-pure nitrogen (>99.999%) and an empty 1 L PEA bag, respectively. A Teflon tube was used to connect the quartz tube and the gas cylinder at one end and the 1 L PEA bag at the other. Then, 1 μL of the 2^nd^ L-WS was injected onto the quartz tube using a temporary injection port (made from a Teflon tube), in order to connect the inlet of the quartz tube and the gas line. The nitrogen gas in the gas cylinder was delivered to the 1 L PEA bag from the reservoir at a rate of 100 mL min^-1^ for 10 min. Subsequently, the quartz trap was heated at 280°C for 10 minutes so as to vaporize all of the target compounds contained in the L-WS.

The GWS used for Experiments 1 and 2 were prepared in a two-step process. Initially, the G-WS with a target concentration level of 225 ± 73.7 ppb (VFAs, phenols, and indoles) was prepared by vaporizing 20 μL of the L-WS (896 ± 381 ng μL^-1^) into a 20 L PEA bag (flow rate = 1000 mL min^-1^ for 20 min). Subsequently, the four reference compounds (BTXS: 100 mL of 20 ppm) were then added into the 20 L bag using commercial standard gas mixtures (RIGAS, Korea). To balance the total volume, we first removed 100 mL of vaporized standard from the bag.

**Quality Assurance/Quality Control (QA/QC) of target compounds**

As part of the QA of the method, the reproducibility, relative standard error (RSE %), and method detection limits (MDL) of the ST-TD-GC-MS were estimated. The RSE values were computed using triplicate analyses of the third calibration point of the L-WS (mean standard deviation (SD) concentration: (1) VFAs, phenols, and indoles 8.96 ± 3.81 ng μL^-1^ and (2) aromatics: 16.4 ± 0.38 ng μL^-1^) loaded onto a ST (sample volume = 1 µL). The MDL values were determined by seven replicate analyses of a L-WS (mean ± SD concentration: (1) VFAs, phenols, and indoles = 0.90 ± 0.38 ng μL^-1^ and (2) aromatics = 0.65 ± 0.02 ng μL^-1^) prepared by two-fold dilution of the first calibration point.

**Instrumental setup**

In this study, the efficiency of the liquid to gas phase transition was evaluated for each target species by estimating the recoveries of the GWS relative to the LWS. The standard samples loaded on the STs were analyzed using gas chromatography (GC, Shimadzu, GC-2010, Japan) and mass spectrometry (MS, Shimadzu GCMS-QP2010, Japan) with a thermal desorber (TD, UNITY II, Markes International, Ltd., UK) system. The TD cryofocusing trap (ID (2mm) and the total sorbent bed length (50 mm)) were packed with an equi-volume ratio of Carbopack-C and -B (Markes International, Ltd., UK) **(Table S2)**. Typically, the standards loaded on the STs were thermally desorbed, transferred to the GC, and separated using a CP-wax column (diameter: 0.25 mm, length: 60 m, and thickness: 0.25 µm) for MS detection. Each target component was initially examined in total ion chromatographic (TIC) mode over a mass range of 35 to 600 m/z with a scan speed of 1250 sec^-1^. Later, to remove the influence of the potential interferences, the mass spectral data of each target compound was identified using extracted ion chromatographic (EIC) mode.

**Synthesis of MOFs and their characterization**

Reagent grade chemicals (zinc nitrate hexahydrate (Zn(NO_3_)_2_∙6H_2_O: 98%), copper nitrate hemi penta-hydrate (Cu(NO_3_)_2_∙2.5H_2_O: ≥ 98%), europium (III) nitrate penta-hydrate (Eu(NO_3_)_3_∙5H_2_O: 99.9%), trimesic acid (H_3_BTC: 95%), terephthalic acid (BDC: 98%), dichloromethane (CH_2_Cl_2_: ≥99.8%), triethylamine ((TEA ≥ 99%), and ethanol (≥ 99.5%)) were purchased from Sigma Aldrich, USA. N,N-dimethylformamide (DMF: 99.0% : Samchun Chemicals, South Korea) and chloroform (CH_3_Cl_3_: ≥99.5% : Daejung Chemicals, South Korea) were also purchased commercially. The commercial adsorbents Tenax TA (60/80 mesh), Carboxen 1000 (60/80 mesh), and Carbopack X (40/60 mesh) were purchased from Supelco Analytical, USA.

**(1) MOF-199:** The blue uniform crystals of the MOF-199 were synthesized by following the procedure of Millward and Yaghi [1]. To begin with, 10 g of Cu(NO_3_)_2_∙2.5H_2_O and 5 g of H_3_BTC were mixed and stirred (Wisestir MSH-20A, Wise Laboratory Instruments, South Korea) for 10 minutes using 250 mL of a solvent containing a 1:1:1 ratio of DMF, ethanol, and water in a 500 mL jar. Later, the slurry was transferred to a tightly capped glass vessel and was heated at 85°C for 20 h (forced convection oven CO-42/81/150, Hanyang Scientific Equipment, South Korea). The obtained blue crystals were washed with 50 mL of DMF and exchanged with CH_2_Cl_2_ thrice in three days. Finally, the dark blue color crystals were filtered and held under drying at 170 ˚C overnight.

**(2) MOF-5:** The synthesis method of MOF-5 was adopted from Kumar et. al.,Kumar, Paul and Deep [2] with some modifications. Specifically, 1.4 g of Zn(NO_3_)_2_∙6H_2_O and 0.3 g of BDC were dissolved in 50 mL of DMF in a 100 mL beaker. Then, 2 mL of TEA was added drop-wise using a micropipette while stirring the above mixture. The resulting slurry was covered and was then stirred continuously for 2 h at room temperature. The slurry was then filtered and exhaustively washed with CHCl_3_ to remove DMF. Finally, it was oven-dried for 12 h at 90 ˚C.

**(3) Eu-MOF:** Nano sized Eu-MOF was synthesized according to the procedure of Choi et al. [3]. To begin with, 2 mL of TEA was added very slowly to 40 mL of a methanol solution containing 400 mg of Eu(NO_3_)_3_∙5H_2_O and 401 mg of BDC in a 100 mL beaker. The mixture was continuously stirred for 2 h at room temperature, and the precipitated solid product was collected by centrifugation, washed with methanol, and then oven-dried for 12 h at 90°C.

**N_2_ adsorption isotherms**

The measured N_2_ adsorption-desorption isotherms was summarized in **Figure S1**. The MOF-5 and MOF-199 showed type-1 adsorption isotherms showing the ordered microporous structure. On the other hand, the unusual type-V adsorption-desorption isotherm was noticed for the Eu-MOF and which suspected due to the ununiformed nanopore distribution of the Eu-MOF. This adsorption-desorption pattern indicates the nano-porous nature of the framework.

**2 Results and discussions**

**Limitation of quartz wool in an air sample analysis**

In this study, we found some limitations in direct estimation of sorbent capacity for certain compounds that have considerably smaller breakthrough volumes (BTVs) on some selected sorbents. Under such circumstances, the apparatus components (e.g., QW and tubing) had relatively more sorption for certain compounds than the selected sorbent. Unfortunately, this was unavoidable as we tried to maintain a minimal amount of sorbents (MOF/adsorbents) to facilitate the quick observation of their BTV by considering the indoor applications. For instance, as the BTV for ACA and PPA on Tenax TA are very small (<http://www.sisweb.com/index/referenc/tenaxta.htm>), their sorption is likely to be affected considerably by sorptive losses to apparatus components (rather than the test sorbent). In principle, the sorption data for the weaker sorbents (such as Eu-MOF and Tenax TA) can be subtracted from the strong adsorbents such as MOF-199 and Carboxen 1000 to estimate net sorption capacities. Instead, we followed the procedure described above using Eqn. (2) to estimate sorptive losses to apparatus components. To this end, our present research provides a valuable insight into the application of MOFs as sorbent to abate the indoor air pollutants at ambient conditions.

**Changes in MOF IR bands due to adsorption:**

The microscopic mechanism of the host-guest interaction was investigated in terms of changes in the chemical structural properties before and after the adsorption of guest molecules on MOFs by infrared spectroscopy (**Figure S3**). The IR spectra of lab synthesized MOF-5, Eu-MOF, and MOF-199 agreed well with literature data [2, 4, 5]. The framework structure of all three MOFs remained intact before and after adsorption. In addition, the FTIR results suggest favorable π-π interactions of the guest molecules with COO^‑^ and C=C regions in the MOFs.

Note that the most important MOF-5 IR peaks were seen at 1502, 1385, 816, 746.4, and 653 cm^-1^ for the C-O-Zn and Zn-O bond vibrations. In addition, the peak at 2989 cm^-1^ indicated a –C-H stretching frequency for the guest molecules. On the other hand, MOF-199 showed a considerable reduction in the frequencies of asymmetric (1640 cm^-1^) and symmetric (1369 cm^-1^) C=C stretching vibrations [6]. The adsorption spectrum of the Eu-MOF also showed a similar pattern to that of the MOF-199, although a very drastic decrease in peaks below 1000 cm^-1^ was observed. However, the smaller migration in the region of 1582 to 1578 cm^-1^ (changes in the C=C region) also indicates the possible π-π interactions between the framework and guest molecules [7]. The findings of similar interaction patterns observed from Eu-MOF to the MOF-199 before and after adsorption suggest the possibility that they may share a similar adsorption mechanism. Although Eu^3+^ has free 4f electrons for interactions (Lanthanide series) due to its complex space orientation of electronic orbitals, the possible metal ion-guest molecule interactions in the space were restricted. Hence, we assumed that the estimated sorption capacity of Eu-MOF was lower than that of MOF-199.

**Table captions**

**Table S1. S**ummary of procedures involved in the preparation of liquid and solid phase standards

(A) Preparation of liquid-phase primary standard for VFAs, phenols, and indoles.

(B) Summary of procedures used for the preparation of liquid-phase working standards for recovery experiment (Experiment 1).

(C) Summary of procedures involved in the preparation of liquid standards for comparative removal analysis for MOF (Experiments 2 and 3).

**Table S2.** Instrumental setups and operational conditions for the analysis of target VOCs using TD-GC-MS.

**Table S3.** Comparison of calibration results for all the different concentration levels of standards in a 1 L PEA bag (Experiment 1).

**Table S4.** Comparison of target compounds prepared by the vaporization of L-WS in 1L PEA bag.

**Table S5.** Comparison of calibration results at each of all different concentration levels of standards in a 20 L PEA bag.

**Table S6**. Comparison of the recovery of target compounds prepared by the vaporization of L-WS into a 20 L PEA bag.

**Table S7.** Henry’s law constants (HLC, mol.kg^-1^.Pa^-1^) and sorbent capacities (Cap, mg.g^-1^, after 15 L loading) for 14 VOCs on 3 MOFs and 3 commercial sorbents and VOC loss factors (LF) to system components

**Table S8.** DFT 0 K adsorption enthalpies and estimated 298 K Gibbs free energies (kJ.mol^-1^) for representative chemical species on two different MOFs examined in this study as compared to other sorbents.

**Table S9.** Preparation of vaporized gaseous samples of BTXS, VFAs, phenols, and indoles and their corresponding concentration levels used for the sorptive removal analysis.

**Figure captions**

**Figure S1.** N_2_ adsorption isotherms of MOF-5, Eu-MOF, and MOF-199

**Figure S2**. Mass sorbed apportioned to mass sorbed on selected sorbent and total sorbed mass (sorbent + losses to system components and Henry's law constant (HLC) versus total mass loaded.

**Figure S3**. FTIR spectra for (a) MOF-5, (b) Eu-MOF, and (c) MOF-199 before and after the adsorption experiment.

**Table S1.** Summary of procedures involved in the preparation of liquid and solid phase standards.

**(A)** Preparation of liquid-phase primary standard for VFAs, phenols, and indoles.

|  |  |  |  | Liquid (μL) |  |  |  | Liquid (μL) |  |  |  |  |  | | Solid (mg) | | | | | | | | | | |
| --- | --- | --- | --- | --- | --- | --- | --- | --- | --- | --- | --- | --- | --- | --- | --- | --- | --- | --- | --- | --- | --- | --- | --- | --- | --- |
| 1 | Standard type |  |  | Aromatic HC |  |  |  |  |  |  |  |  |  | | Phenols and indoles | | | | | | | | | | |
|  |  |  |  | B | T | p-X | S | ACA | PPA | IBA | BTA | IVA | VLA | | PhAl | | p-C | | ID | | | SK | | |  |
| 2 | Purity of reagent grade chemicals (%) | |  |  |  |  |  | 99.0 | 99.0 | 99.0 | 99.0 | 99.0 | 99.0 | | 99.0 | | 99.0 | | | | 99.0 | | | 98.0 | |
| 3 | Primary standard (PS) used to prepare gaseous working standard (G-WS) (Exp-1 and Exp -2) | | | | | | |  |  |  |  |  |  | |  | |  | | | |  | | |  | |
|  | (A) PS-1 Dissolution of solid phase chemicals | | |  |  |  |  |  |  |  |  |  | |  | | 601 | | 601 | | 603 | | | 603 | | |
|  | (B) PS-2 Mixing of liquid phase chemicals | | |  |  |  |  | 1,000 | 1,000 | 1,000 | 1,000 | 1,000 | | 1,000 | |  | |  | |  | | |  | | |
|  | (C) PS-3 Mixing of liquid phase chemicals | | | 500 | 500 | 500 | 500 |  |  |  |  |  | |  | |  | |  | |  | | |  | | |
|  |  |  |  |  |  |  |  |  |  |  |  |  |  | |  | |  | |  | | |  | | |  |
| 4 | Concentration (ng μL^-1^) | |  | 21,803 | 21,641 | 21,310 | 22,498 | 51,926 | 49,005 | 48,000 | 47,520 | 46,035 | 46,530 | | 26,798 | | 26,785 | | | | 26,865 | | | 26,593 | |

**(B)** Summary of procedures used for the preparation of liquid-phase working standards for recovery experiment (Experiment 1).

|  | Compounds | |  | First L-WS^c^ | |  |  | Second L-WS^d^ (ng μL^-1^) | | |  |  |  |  |  | Final L-WS^e^ (ng μL^-1^) | | |  |  |  |  | G-WS^f^ (ppb) | |  |
| --- | --- | --- | --- | --- | --- | --- | --- | --- | --- | --- | --- | --- | --- | --- | --- | --- | --- | --- | --- | --- | --- | --- | --- | --- | --- |
|  |  | |  | (ng µL^-1^) |  |  | 1st | 2nd | 3rd | 4th |  |  | 1st | 2nd | 3rd | 4th | 5th | 6^th^ | 7th | 8th |  | 1st | 2nd | 3rd | 4th |
| 1 | ACA | |  | 1,298 |  |  | 130 | 260 | 649 | 1298 |  |  | 1.30 | 2.60 | 6.49 | 13.0 | 26.0 | 51.9 | 104 | 130 |  | 52.9 | 106 | 264 | 529 |
| 2 | PPA | |  | 1,225 |  |  | 123 | 245 | 613 | 1225 |  |  | 1.23 | 2.45 | 6.13 | 12.3 | 24.5 | 49.0 | 98.0 | 123 |  | 40.4 | 80.9 | 202 | 404 |
| 3 | IBA | |  | 1,200 |  |  | 120 | 240 | 600 | 1200 |  |  | 1.20 | 2.40 | 6.00 | 12.0 | 24.0 | 48.0 | 96.0 | 120 |  | 33.3 | 66.6 | 167 | 333 |
| 4 | BTA | |  | 1,188 |  |  | 119 | 238 | 594 | 1188 |  |  | 1.19 | 2.38 | 5.94 | 11.9 | 23.8 | 47.5 | 95.0 | 119 |  | 33.0 | 65.9 | 165 | 330 |
| 5 | IVA | |  | 1,151 |  |  | 115 | 230 | 575 | 1151 |  |  | 1.15 | 2.30 | 5.75 | 11.5 | 23.0 | 46.0 | 92.1 | 115 |  | 27.6 | 55.1 | 138 | 276 |
| 6 | VLA | |  | 1,163 |  |  | 116 | 233 | 582 | 1163 |  |  | 1.16 | 2.33 | 5.82 | 11.6 | 23.3 | 46.5 | 93.1 | 116 |  | 27.9 | 55.7 | 139 | 279 |
| 7 | PhAl | |  | 2,680 |  |  | 268 | 536 | 1340 | 2680 |  |  | 2.68 | 5.36 | 13.4 | 26.8 | 53.6 | 107 | 214 | 268 |  | 69.6 | 139 | 348 | 696 |
| 8 | p-C | |  | 2,678 |  |  | 268 | 536 | 1339 | 2678 |  |  | 2.68 | 5.36 | 13.4 | 26.8 | 53.6 | 107 | 214 | 268 |  | 60.6 | 121 | 303 | 606 |
| 9 | ID | |  | 2,686 |  |  | 269 | 537 | 1343 | 2686 |  |  | 2.69 | 5.37 | 13.4 | 26.9 | 53.7 | 107 | 215 | 269 |  | 56.1 | 112 | 280 | 561 |
| 10 | SK | |  | 2,659 |  |  | 266 | 532 | 1330 | 2659 |  |  | 2.66 | 5.32 | 13.3 | 26.6 | 53.2 | 106 | 213 | 266 |  | 49.6 | 99.1 | 248 | 496 |
| Mixing recipe in | | | PS-1^a^ | 250 |  | 1^st^ L-WS | 1,000 | 400 | 1,000 | 2,000 |  | 1st cal. point of the | 20 | 40 | 100 | 200 | 400 | 800 | 1,600 | 2,000 |  |  |  |  |  |
| volume (μL)^b^ | | | PS-2^a^ | 1,000 |  |  |  |  |  |  |  | second L-WS |  |  |  |  |  |  |  |  |  |  |  |  |  |
|  | |  | MeOH | 8,750 |  | MeOH | 9,000 | 1,600 | 1,000 | 0 |  |  | 1,980 | 1,960 | 1,900 | 1,800 | 1,600 | 1,200 | 400 | 0 |  |  |  |  |  |
|  | |  | Total | 10,000 |  | Total | 10,000 | 2,000 | 2,000 | 2,000 |  |  | 2,000 | 2,000 | 2,000 | 2,000 | 2,000 | 2,000 | 2,000 | 2,000 |  |  |  |  |  |

^a^Primary standard (PS) used to make the liquid working standard.

^b^Mixing conditions used for L-WS preparation.

^c^The first liquid working standard (1^st^ L-WS): mixture of PS-1, PS-2, and dilution of PS mixture using methanol.

^d^The second liquid working standard (2^nd^ L-WS): dilution of the first L-WS using methanol.

The second L-WS was used to prepare the gaseous working standard (G-WS).

^e^The final liquid working standard (Final L-WS): dilution of the first point of the second L-WS using methanol.

^f^Theoretical concentration of gaseous working standard (G-WS). The second L-WS was vaporized at 280°C and swept into a 1 L polyester aluminum (PEA) bag.

The mixing ratios were computed under the assumption that all liquid was converted into the gas phase standard without any loss.

Vaporization parameter: (1) nitrogen (>99.999%), (2) flow rate: 100 mL min-1, (3) sweep time: 10 min, and sample volume: 1 L.

**(C)** Summary of procedures involved in the preparation of liquid standards for comparative removal analysis for MOF (Experiments 2 and 3).

| **Order** | **Compounds** |  | **First L-WS** |  |  |  | **Second L-WS** | |  |  |  |  |  | **Final L-WS (ng μL^-1^)** | | |  |  |  | **G-WS^a^** |
| --- | --- | --- | --- | --- | --- | --- | --- | --- | --- | --- | --- | --- | --- | --- | --- | --- | --- | --- | --- | --- |
|  |  |  | (ng µL^-1^) |  |  |  | (ng μL^-1^) |  |  |  | 1st | 2nd | 3rd | 4th | 5th | 6th | 7th | 8th |  |  |
| 5 | ACA |  | 1,298 |  |  |  | 649 |  |  |  | 6.49 | 13.0 | 32.5 | 64.9 | 130 | 260 | 325 | 389 |  | 264 |
| 6 | PPA |  | 1,225 |  |  |  | 613 |  |  |  | 6.13 | 12.3 | 30.6 | 61.3 | 123 | 245 | 306 | 368 |  | 202 |
| 7 | IBA |  | 1,200 |  |  |  | 600 |  |  |  | 6.00 | 12.0 | 30.0 | 60.0 | 120 | 240 | 300 | 360 |  | 167 |
| 8 | BTA |  | 1,188 |  |  |  | 594 |  |  |  | 5.94 | 11.9 | 29.7 | 59.4 | 119 | 238 | 297 | 356 |  | 165 |
| 9 | IVA |  | 1,151 |  |  |  | 575 |  |  |  | 5.75 | 11.5 | 28.8 | 57.5 | 115 | 230 | 288 | 345 |  | 138 |
| 10 | VLA |  | 1,163 |  |  |  | 582 |  |  |  | 5.82 | 11.6 | 29.1 | 58.2 | 116 | 233 | 291 | 349 |  | 139 |
| 11 | PhAl |  | 2,680 |  |  |  | 1,340 |  |  |  | 13.4 | 26.8 | 67.0 | 134 | 268 | 536 | 670 | 804 |  | 348 |
| 12 | p-C |  | 2,678 |  |  |  | 1,339 |  |  |  | 13.4 | 26.8 | 67.0 | 134 | 268 | 536 | 670 | 804 |  | 303 |
| 13 | ID |  | 2,686 |  |  |  | 1,343 |  |  |  | 13.4 | 26.9 | 67.2 | 134 | 269 | 537 | 672 | 806 |  | 280 |
| 14 | SK |  | 2,659 |  |  |  | 1,330 |  |  |  | 13.3 | 26.6 | 66.5 | 133 | 266 | 532 | 665 | 798 |  | 248 |
| 1 | B |  |  | 21,803 |  |  |  | 327 |  |  | 3.27 | 6.54 | 16.4 | 32.7 | 65.4 | 131 | 262 | 327 |  | 102^b^ |
| 2 | T |  |  | 21,641 |  |  |  | 325 |  |  | 3.25 | 6.49 | 16.2 | 32.5 | 64.9 | 130 | 260 | 325 |  | 101^b^ |
| 3 | p-X |  |  | 21,310 |  |  |  | 320 |  |  | 3.20 | 6.39 | 16.0 | 32.0 | 63.9 | 128 | 256 | 320 |  | 100^b^ |
| 4 | S |  |  | 22,498 |  |  |  | 337 |  |  | 3.37 | 6.75 | 16.9 | 33.7 | 67.5 | 135 | 270 | 337 |  | 102^b^ |
| Mixing recipe in | | PS-1 | 250 |  |  | First L-WS | 5,000 | 150 |  | Second L-WS | 20 | 40 | 100 | 200 | 400 | 800 | 1,600 | 2,000 |  |  |
| volume (μL) | | PS-2 | 1,000 |  |  |  |  |  |  |  |  |  |  |  |  |  |  |  |  |  |
|  |  | PS-3 |  | 5,000 |  |  |  |  |  |  |  |  |  |  |  |  |  |  |  |  |
|  |  | MeOH | 18,750 | 15,000 |  | MeOH | 5,000 | 9,850 |  | MeOH | 1,980 | 1,960 | 1,900 | 1,800 | 1,600 | 1,200 | 400 | 0 |  |  |
|  |  | Total | 20,000 | 20,000 |  | Total | 10,000 | 10,000 |  | Total | 2000 | 2000 | 2000 | 2000 | 2000 | 2000 | 2000 | 2000 |  |  |

^a^The G-WS of carboxyl, phenol, and indoles were prepared by vaporizing the L-WS.

The second liquid working standard (2^nd^ L-WS): dilution of the first L-WS using methanol.

Theoretical concentration of gaseous working standard (G-WS). The second L-WS was vaporized at 280°C and swept into a 20 L polyester aluminum (PEA) bag.

The mixing ratios were computed under the assumption that all liquid was converted into the gas phase standard without any loss.

Vaporization parameter: (1) nitrogen (>99.999%), (2) flow rate: 1000 mL min^-1^, (3) sweep time: 20 min, and sample volume: 20 L.

^b^The gaseous working standards of aromatics were prepared by purging out 100 mL of the prepared G-WS in a 20 L bag using a 100 mL syringe and 100 mL of the 20 ppm (B = 20.3, T = 20.0, p-X = 19.9 and S = 20.2) G-WS was added to the 20 L bag.

**Table S2.** Instrumental setups and operational conditions for the analysis of target VOCs using TD-GC-MS.

| **(A) GC (SHIMADZU GC-2010, JAPAN), MS (SHIMADZU GCMS-QP2010, JAPAN)** | | | | |
| --- | --- | --- | --- | --- |
| Column: CP Wax (diameter: 0.25 mm, length: 30 m, and film thickness: 0.25 µm) | | | | |
| **(1) Oven setting** | |  | **(2) Detector setting** | |
| Oven temp: | 40 °C (5 min) |  | Ionization mode: | EI (70eV) |
| Oven rate: | 20 °C min^-1^ (9 min) |  | Ion source temp: | 230 °C |
| Max oven temp: | 220 °C (16 min) |  | Interface temp: | 230 °C |
| Total time: | 30 min |  | TIC scan range: | 35-600 m z^-1^ |
|  |  |  | Scan speed | 1250 sec^-1^ |
| **(3) Carrier gas setting** |  |  |  |  |
| Gas type: | He (>99.999%) |  | Column flow: | 1.03 mL min^-1^ |
| Constant gas pressure | 16.0 psi |  |  |  |
| **(B) Thermal desorber (TD) (UNITY II, MARKES INTERNATIONAL, LTD., UK).** | | | | |
| Cold trap sorbent: | Quartz wool + Carbopack C + Carbopack B (Volume ratio = 1:1:1) | | | |
| Split ratio: | 0.103 |  | Adsorption temp: | -10 °C |
| Split flow: | 10 mL |  | Desorption temp: | 300 °C |
| Trap hold time: | 10 min |  | Flow path temp: | 150 °C |
| **(C) Sorbent (Sampling) Tube** | | | |  |
| Sorbent material: | Carbopack C + Carbopack B + Carbopack X (70, 50, and 50 mg) , Quartz wool (20 mg) | | | |
| Desorption flow: | 100 mL min^-1^ |  |  |  |
| Desorption time: | 7 min |  | Desorption temp: | 300 °C |
| **(D) Information about sorbent tube sampling approach.** | | | | |
| Calibration of liquid working standard | |  |  |  |
| a. Working standard phase: | Liquid |  |  |  |
| b. Injection volume: | 1 | μL |  |  |
| c. Sweep gas | N_2_ (>99.999%) |  |  |  |
| d. Sweep flow rate: | 100 | mL min-1 |  |  |
| e. Sweeping time: | 3 | min |  |  |
| f. Sweep loading volume: | 300 | mL |  |  |

**Table S3.** Comparison of calibration results at each different concentration level of the standards in 1 L PEA bag (Experiment 1).

| Exp |  | | VFAs |  |  |  |  |  |  | Phenols |  |  | Indoles |  |
| --- | --- | --- | --- | --- | --- | --- | --- | --- | --- | --- | --- | --- | --- | --- |
| code |  | | ACA | PPA | IBA | BTA | IVA | VLA |  | PhAl | p-C |  | ID | SK |
| **A. Direct injection of the L-WS** | | | |  |  |  |  |  |  |  |  |  |  |  |
| [1] Response factor (ng^-1^) | | |  |  |  |  |  |  |  |  |  |  |  |  |
|  |  | | 6,092 | 3,744 | 5,803 | 14,649 | 16,730 | 16,727 |  | 23,036 | 20,311 |  | 30,479 | 34,293 |
| [2] Coefficient of determination (R^2^) | | | |  |  |  |  |  |  |  |  |  |  |  |
|  |  | | 0.9909 | 0.9983 | 0.9941 | 0.9943 | 0.996 | 0.995 |  | 0.9982 | 0.9988 |  | 0.9970 | 0.9968 |
| **B. Exp 1: Direct injection of the G-WS** | | | |  |  |  |  |  |  |  |  |  |  |  |
| [1] Response factor (ng^-1^) | | |  |  |  |  |  |  |  |  |  |  |  |  |
| ***I. FSC - 1*** | |  |  |  |  |  |  |  |  |  |  |  |  |  |
| a. FSC-1 (A) | |  | 2,582 | 2,206 | 3,682 | 7,652 | 10,562 | 8,366 |  | 5,975 | 4,643 |  | 2,487 | 3,311 |
| b. FSC-1 (B) | |  | 3,264 | 2,148 | 3,975 | 7,336 | 11,584 | 7,796 |  | 6,744 | 5,061 |  | 2,868 | 3,573 |
|  | | Mean | 2,923 | 2,177 | 3,828 | 7,494 | 11,073 | 8,081 |  | 6,360 | 4,852 |  | 2,677 | 3,442 |
|  | | SD | 482 | 40.9 | 207 | 224 | 723 | 403 |  | 544 | 296 |  | 270 | 185 |
|  | | RSE (%) | 11.7 | 1.33 | 3.82 | 2.11 | 4.61 | 3.53 |  | 6.05 | 4.31 |  | 7.13 | 3.79 |
| ***II. FSC - 2*** | |  |  |  |  |  |  |  |  |  |  |  |  |  |
| a. FSC-2 (A) | |  | 2,875 | 1,880 | 3,377 | 6,804 | 8,270 | 7,189 |  | 6,772 | 3,925 |  | 2,535 | 3,249 |
| b. FSC-2 (B) | |  | 2,519 | 1,810 | 3,121 | 6,443 | 8,307 | 6,953 |  | 5,591 | 4,379 |  | 2,328 | 2,881 |
|  | | Mean | 2,697 | 1,845 | 3,249 | 6,624 | 8,289 | 7,071 |  | 6,181 | 4,152 |  | 2,432 | 3,065 |
|  | | SD | 252 | 49.2 | 181 | 255 | 26.6 | 166 |  | 835 | 321 |  | 147 | 261 |
|  | | RSE (%) | 6.59 | 1.89 | 3.95 | 2.72 | 0.23 | 1.66 |  | 9.56 | 5.46 |  | 4.26 | 6.01 |
| ***III. FSC - 3*** | |  |  |  |  |  |  |  |  |  |  |  |  |  |
| a. FSC-3 (A) | |  | 1,984 | 2,453 | 3,731 | 8,443 | 6,962 | 8,977 |  | 4,533 | 3,540 |  | 1,823 | 2,373 |
| b. FSC-3 (B) | |  | 2,137 | 1,970 | 3,291 | 7,140 | 7,659 | 7,577 |  | 4,775 | 4,778 |  | 2,327 | 3,067 |
|  | | Mean | 2,061 | 2,211 | 3,511 | 7,792 | 7,311 | 8,277 |  | 4,654 | 4,159 |  | 2,075 | 2,720 |
|  | | SD | 109 | 342 | 311 | 921 | 493 | 990 |  | 171 | 875 |  | 356 | 491 |
|  | | RSE (%) | 3.73 | 10.9 | 6.26 | 8.36 | 4.77 | 8.45 |  | 2.61 | 14.9 |  | 12.1 | 12.8 |
| ***III. FSC - 4*** | |  |  |  |  |  |  |  |  |  |  |  |  |  |
| a. FSC-4 (A) | |  | 2,590 | 2,555 | 3,483 | 7,496 | 9,528 | 7,959 |  | 8,042 | 5,377 |  | 3,693 | 3,410 |
| b. FSC-4 (B) | |  | 2,511 | 2,324 | 3,499 | 7,787 | 9,114 | 8,091 |  | 7,877 | 5,851 |  | 3,133 | 3,230 |
|  | | Mean | 2,551 | 2,439 | 3,491 | 7,641 | 9,321 | 8,025 |  | 7,960 | 5,614 |  | 3,413 | 3,320 |
|  | | SD | 55.9 | 163 | 11.2 | 206 | 292 | 93 |  | 117 | 335 |  | 397 | 127 |
|  | | RSE (%) | 1.55 | 4.73 | 0.23 | 1.90 | 2.22 | 0.82 |  | 1.04 | 4.22 |  | 8.22 | 2.71 |
|  | | Total mean (n=8) | 2,558 | 2,168 | 3,520 | 7,387 | 8,998 | 7,863 |  | 6,289 | 4,694 |  | 2,649 | 3,137 |
|  | | SD | 398 | 269 | 270 | 615 | 1529 | 646 |  | 1309 | 753 |  | 573 | 372 |
|  | | RSE (%) | 5.51 | 4.39 | 2.72 | 2.95 | 6.01 | 2.91 |  | 7.36 | 5.67 |  | 7.65 | 4.20 |
| [2] Coefficient of determination | | |  |  |  |  |  |  |  |  |  |  |  |  |
| ***I. FSC - 1*** | |  |  |  |  |  |  |  |  |  |  |  |  |  |
| a. FSC-1 (A) | |  | 0.9621 | 0.9979 | 0.9959 | 0.9985 | 0.9956 | 0.9961 |  | 0.9885 | 0.9858 |  | 0.9919 | 0.9915 |
| b. FSC-1 (B) | |  | 0.9911 | 0.9892 | 0.9914 | 0.9924 | 0.998 | 0.9951 |  | 0.9948 | 0.9966 |  | 0.9972 | 0.9947 |
| ***II. FSC - 2*** | |  |  |  |  |  |  |  |  |  |  |  |  |  |
| a. FSC-2 (A) | |  | 0.9949 | 0.9901 | 0.9947 | 0.992 | 0.9961 | 0.991 |  | 0.9744 | 0.9825 |  | 0.9891 | 0.9861 |
| b. FSC-2 (B) | |  | 0.9932 | 0.9941 | 0.9984 | 0.997 | 0.9952 | 0.9952 |  | 0.9968 | 0.9968 |  | 0.9963 | 0.9958 |
| ***III. FSC - 3*** | |  |  |  |  |  |  |  |  |  |  |  |  |  |
| a. FSC-3 (A) | |  | 0.9987 | 0.9959 | 0.9956 | 0.9953 | 0.9992 | 0.9926 |  | 0.9907 | 0.99 |  | 0.9855 | 0.9948 |
| b. FSC-3 (B) | |  | 0.9916 | 0.9945 | 0.9964 | 0.9975 | 0.9987 | 0.9987 |  | 0.9979 | 0.9979 |  | 0.9846 | 0.9968 |
| ***III. FSC - 4*** | |  |  |  |  |  |  |  |  |  |  |  |  |  |
| a. FSC-4 (A) | |  | 0.9841 | 0.9915 | 0.9889 | 0.9906 | 0.9883 | 0.9909 |  | 0.9925 | 0.9896 |  | 0.9929 | 0.9859 |
| b. FSC-4 (B) | |  | 0.9573 | 0.9932 | 0.9945 | 0.995 | 0.9954 | 0.9942 |  | 0.9947 | 0.9945 |  | 0.9893 | 0.9939 |

* (A) and (B) in parentheses indicate that the vaporization was conducted two times consecutively (duplicate analyses).

**Table S4.** Comparison of target compounds prepared by the vaporization of L-WS in 1 L PEA bag.

| Compound | Percentage difference of Exp 1 (%)^a^ | | | |  |  |  |  | Relative recovery (%)^b^ | | |  |  |  |  |  | Actual concentration (ppb)^c^ | | |  |
| --- | --- | --- | --- | --- | --- | --- | --- | --- | --- | --- | --- | --- | --- | --- | --- | --- | --- | --- | --- | --- |
|  | FSC-1 | FSC-2 | FSC-3 | FSC-4 |  | Mean | RSE (%) |  | FSC-1 | FSC-2 | FSC-3 | FSC-4 |  | Mean | RSE (%) |  | FSC-1 | FSC-2 | FSC-3 | FSC-4 |
| **A. VFAs** |  |  |  |  |  |  |  |  |  |  |  |  |  |  |  |  |  |  |  |  |
| ACA | 52.0 | 55.7 | 66.2 | 58.1 |  | 58.0 | 4.47 |  | 48.0 | 44.3 | 33.8 | 41.9 |  | 42.0 | 6.18 |  | 25.4 | 46.8 | 89.4 | 221 |
| PPA | 41.9 | 50.7 | 40.9 | 34.8 |  | 42.1 | 6.73 |  | 58.1 | 49.3 | 59.1 | 65.2 |  | 57.9 | 4.89 |  | 23.5 | 39.9 | 119 | 263 |
| IBA | 34.0 | 44.0 | 39.5 | 39.8 |  | 39.3 | 4.51 |  | 66.0 | 56.0 | 60.5 | 60.2 |  | 60.7 | 2.92 |  | 22.0 | 37.3 | 101 | 200 |
| BTA | 48.8 | 54.8 | 46.8 | 47.8 |  | 49.6 | 3.12 |  | 51.2 | 45.2 | 53.2 | 52.2 |  | 50.4 | 3.07 |  | 16.9 | 29.8 | 87.7 | 172 |
| IVA | 33.8 | 50.5 | 56.3 | 44.3 |  | 46.2 | 9.01 |  | 66.2 | 49.5 | 43.7 | 55.7 |  | 53.8 | 7.74 |  | 18.2 | 27.3 | 60.2 | 154 |
| VLA | 51.7 | 57.7 | 50.5 | 52.0 |  | 53.0 | 2.63 |  | 48.3 | 42.3 | 49.5 | 48.0 |  | 47.0 | 2.97 |  | 13.5 | 23.5 | 68.9 | 134 |
| **B. Phenols** |  |  |  |  |  |  |  |  |  |  |  |  |  |  |  |  |  |  |  |  |
| PhAl | 72.4 | 73.2 | 79.8 | 65.4 |  | 72.7 | 3.49 |  | 27.6 | 26.8 | 20.2 | 34.6 |  | 27.3 | 9.31 |  | 19.2 | 37.4 | 70.3 | 241 |
| p-C | 76.1 | 79.6 | 79.5 | 72.4 |  | 76.9 | 1.93 |  | 23.9 | 20.4 | 20.5 | 27.6 |  | 23.1 | 6.41 |  | 14.5 | 24.8 | 62.0 | 167 |
| **C. Indoles** |  |  |  |  |  |  |  |  |  |  |  |  |  |  |  |  |  |  |  |  |
| ID | 91.2 | 92.0 | 93.2 | 88.8 |  | 91.3 | 0.88 |  | 8.78 | 7.98 | 6.81 | 11.20 |  | 8.69 | 9.25 |  | 4.93 | 8.95 | 19.1 | 62.8 |
| SK | 90.0 | 91.1 | 92.1 | 90.3 |  | 90.9 | 0.44 |  | 10.0 | 8.94 | 7.93 | 9.68 |  | 9.15 | 4.41 |  | 4.98 | 8.86 | 19.7 | 48.0 |

^a^Percentage difference =(RF value of L-WS-RF value of G-WS/RF value of L-WS) × 100.

^b^Relative recovery = RF value of G-WS/RF value of L-WS × 100.

^c^Actual concentration (ppb) = Ideal concentration of G-WS × Relative recovery (%) / 100.

FSC (Fixed standard concentration): analysis of 10, 20, 50, and 100 mL of the prepared G-WS at 45.1 ppb (FSC-1), 90.2 ppb (FSC-2), 225 ppb (FSC-3), and 451 ppb (FSC-4).

**Table S5.** Comparison of calibration results at all different concentration levels of standards in 20 L PEA bag.

| Exp | |  | Carboxyl | |  |  |  |  |  | Phenol |  |  | Indole |  |
| --- | --- | --- | --- | --- | --- | --- | --- | --- | --- | --- | --- | --- | --- | --- |
| Code | |  | ACA | PPA | IBA | BTA | IVA | VLA |  | PhAl | p-C |  | ID | SK |
| **A. Direct injection of the L-WS** | | | |  |  |  |  |  |  |  |  |  |  |  |
| [1] Response factor (ng^-1^) | | | |  |  |  |  |  |  |  |  |  |  |  |
|  |  | | 6092.4 | 3744.1 | 5802.9 | 14649 | 16730 | 16727 |  | 23036 | 20311 |  | 30479 | 34293 |
| [2] Coefficient of determination (R^2^) | | | | |  |  |  |  |  |  |  |  |  |  |
|  | |  | 0.9909 | 0.9983 | 0.9941 | 0.9943 | 0.996 | 0.995 |  | 0.9982 | 0.9988 |  | 0.9970 | 0.9968 |
| **B. Exp 1: Direct injection of the G-WS: Determination of relative recovery in 20 L bag.** | | | | | | | | | | | |  |  |  |
| [1] Response factor (ng^-1^) | | | |  |  |  |  |  |  |  |  |  |  |  |
| ***I. FSC - 1*** | |  |  |  |  |  |  |  |  |  |  |  |  |  |
| a. FSC-1 (A) | |  | 3681 | 1783 | 3149 | 6755 | 9293 | 8363 |  | 11272 | 9261 |  | 6846 | 5391 |
| b. FSC-1 (B) | |  | 3979 | 1583 | 2788 | 6108 | 8542 | 7047 |  | 10351 | 9136 |  | 6548 | 5995 |
|  | | Mean | 3830 | 1683 | 2968 | 6431 | 8918 | 7705 |  | 10812 | 6697 |  | 6697 | 5693 |
|  | | SD | 211 | 141.5 | 255 | 458 | 531 | 931 |  | 651 | 211 |  | 211 | 427 |
|  | | RSE (%) | 3.90 | 5.94 | 6.08 | 5.04 | 4.21 | 8.54 |  | 4.26 | 2.22 |  | 2.22 | 5.30 |
| ***II. FSC - 2*** | |  |  |  |  |  |  |  |  |  |  |  |  |  |
| a. FSC-2 (A) | |  | 2574 | 1448 | 2470 | 5139 | 6462 | 5077 |  | 8842 | 6958 |  | 3983 | 2689 |
| b. FSC-2 (B) | |  | 2553 | 1564 | 2493 | 5707 | 7444 | 6231 |  | 9111 | 8003 |  | 5085 | 3952 |
|  | | Mean | 2564 | 1506 | 2482 | 5423 | 6953 | 5654 |  | 8977 | 7481 |  | 4534 | 3321 |
|  | | SD | 15 | 82 | 16 | 402 | 694 | 816 |  | 190 | 739 |  | 779 | 894 |
|  | | RSE (%) | 0.41 | 3.84 | 0.46 | 5.24 | 7.06 | 10.20 |  | 1.50 | 6.99 |  | 12.15 | 19.03 |
| ***III. FSC - 3*** | |  |  |  |  |  |  |  |  |  |  |  |  |  |
| a. FSC-3 (A) | |  | 2343 | 1982 | 3207 | 7125 | 8658 | 7424 |  | 11306 | 9640 |  | 6600 | 4467 |
| b. FSC-3 (B) | |  | 2587 | 1717 | 2793 | 6653 | 7648 | 7567 |  | 10481 | 10486 |  | 7741 | 5930 |
|  | | Mean | 2061 | 2211 | 3511 | 7792 | 7311 | 8277 |  | 4654 | 4159 |  | 2075 | 2720 |
|  | | SD | 109 | 342 | 311 | 921 | 493 | 990 |  | 171 | 875 |  | 356 | 491 |
|  | | RSE (%) | 3.73 | 10.9 | 6.26 | 8.36 | 4.77 | 8.45 |  | 2.61 | 14.9 |  | 12.1 | 12.8 |
| ***III. FSC - 4*** | |  |  |  |  |  |  |  |  |  |  |  |  |  |
| a. FSC-4 (A) | |  | 2251 | 1943 | 3293 | 7273 | 8440 | 7633 |  | 12494 | 10406 |  | 8345 | 5590 |
| b. FSC-4 (B) | |  | 1681 | 2055 | 2836 | 6975 | 8602 | 7963 |  | 12626 | 10998 |  | 9183 | 6651 |
|  | |  |  |  |  |  |  |  |  |  |  |  |  |  |
|  | | Mean | 1966 | 1999 | 3064 | 7124 | 8521 | 7798 |  | 12560 | 10702 |  | 8764 | 6121 |
|  | | SD | 403 | 79 | 323 | 211 | 115 | 233 |  | 93 | 419 |  | 592 | 750 |
|  | | RSE (%) | 14.50 | 2.79 | 7.46 | 2.09 | 0.95 | 2.11 |  | 0.53 | 2.77 |  | 4.78 | 8.67 |
|  | | Total mean (n=2×4=8) | 2706 | 1760 | 2879 | 6467 | 8136 | 7163 |  | 10810 | 9361 |  | 6791 | 5083 |
|  | | SD | 757 | 220 | 313 | 749 | 895 | 1053 |  | 1397 | 1352 |  | 1687 | 1296 |
|  | | RSE (%) | 9.90 | 4.42 | 3.84 | 4.10 | 3.89 | 5.20 |  | 4.57 | 5.11 |  | 8.78 | 9.01 |
| **[2] Coefficient of determination** | | | |  |  |  |  |  |  |  |  |  |  |  |
| ***I. FSC - 1*** | |  |  |  |  |  |  |  |  |  |  |  |  |  |
| a. FSC-1 (A) | |  | 0.9863 | 0.994 | 0.998 | 0.9942 | 0.9926 | 0.992 |  | 0.9976 | 0.9919 |  | 0.9935 | 0.9847 |
| b. FSC-1 (B) | |  | 0.9824 | 0.9868 | 0.9962 | 0.9949 | 0.9955 | 0.996 |  | 0.9911 | 0.9949 |  | 0.9908 | 0.9926 |
| ***II. FSC - 2*** | |  |  |  |  |  |  |  |  |  |  |  |  |  |
| a. FSC-2 (A) | |  | 0.9878 | 0.9975 | 0.9942 | 0.994 | 0.9921 | 0.99 |  | 0.9928 | 0.989 |  | 0.9904 | 0.9953 |
| b. FSC-2 (B) | |  | 0.9981 | 0.9931 | 0.9906 | 0.9994 | 0.993 | 0.995 |  | 0.9901 | 0.9908 |  | 0.9943 | 0.9945 |
| ***III. FSC - 3*** | |  |  |  |  |  |  |  |  |  |  |  |  |  |
| a. FSC-3 (A) | |  | 0.9895 | 0.9954 | 0.9976 | 0.9967 | 0.9975 | 0.996 |  | 0.994 | 0.9914 |  | 0.9869 | 0.9932 |
| b. FSC-3 (B) | |  | 0.9608 | 0.9975 | 0.9973 | 0.9969 | 0.9863 | 0.985 |  | 0.9923 | 0.9923 |  | 0.9907 | 0.9886 |
| ***III. FSC - 4*** | |  |  |  |  |  |  |  |  |  |  |  |  |  |
| a. FSC-4 (A) | |  | 0.9925 | 0.997 | 0.9911 | 0.9946 | 0.9984 | 0.997 |  | 0.9953 | 0.9963 |  | 0.9833 | 0.9943 |
| b. FSC-4 (B) | |  | 0.9735 | 0.9813 | 0.9971 | 0.9983 | 0.9985 | 0.996 |  | 0.9973 | 0.9967 |  | 0.9939 | 0.9963 |

* (A) and (B) in parentheses indicate that the vaporization was conducted consecutively two times (duplicate analyses).

**Table S6**. Comparison of the recovery of target compounds prepared by the vaporization of L-WS into 20 L PEA bag.

| **Compound** | **Percentage difference in 20 L G-WS (%)^a^** | | | | | |  |  | **Relative recovery for 20 L G-Ws (%)^b^** | | | | | |  |  | **Actual concentration in 20 L G-WS (ppb)^c^** | | | |
| --- | --- | --- | --- | --- | --- | --- | --- | --- | --- | --- | --- | --- | --- | --- | --- | --- | --- | --- | --- | --- |
|  | FSC-1 | FSC-2 | FSC-3 | FSC-4 |  | Mean | RSE (%) |  | FSC-1 | FSC-2 | FSC-3 | FSC-4 |  | Mean | RSE (%) |  | FSC-1 | FSC-2 | FSC-3 | FSC-4 |
|  |  |  |  |  |  |  |  |  |  |  |  |  |  |  |  |  |  |  |  |  |
| ACA | 37.1 | 57.9 | 59.5 | 67.7 |  | 55.6 | 10.1 |  | 62.9 | 42.1 | 40.5 | 32.3 |  | 44.4 | 12.7 |  | 33.2 | 44.5 | 106.9 | 170.6 |
| PPA | 55.0 | 59.8 | 50.6 | 46.6 |  | 53.0 | 4.64 |  | 45.0 | 40.2 | 49.4 | 53.4 |  | 47.0 | 5.23 |  | 18.2 | 32.5 | 99.9 | 215.9 |
| IBA | 48.8 | 57.2 | 48.3 | 47.2 |  | 50.4 | 3.96 |  | 51.2 | 42.8 | 51.7 | 52.8 |  | 49.6 | 4.03 |  | 17.0 | 28.5 | 86.1 | 175.9 |
| BTA | 56.1 | 63.0 | 53.0 | 51.4 |  | 55.9 | 3.98 |  | 43.9 | 37.0 | 47.0 | 48.6 |  | 44.1 | 5.04 |  | 14.5 | 24.4 | 77.5 | 160.3 |
| IVA | 46.7 | 58.4 | 51.3 | 49.1 |  | 51.4 | 4.27 |  | 53.3 | 41.6 | 48.7 | 50.9 |  | 48.6 | 4.52 |  | 14.7 | 22.9 | 67.1 | 140.3 |
| VLA | 53.9 | 66.2 | 55.2 | 53.4 |  | 57.2 | 4.59 |  | 46.1 | 33.8 | 44.8 | 46.6 |  | 42.8 | 6.13 |  | 12.8 | 18.8 | 62.4 | 129.8 |
|  |  |  |  |  |  |  |  |  |  |  |  |  |  |  |  |  |  |  |  |  |
| PhAl | 53.1 | 61.0 | 52.7 | 45.5 |  | 53.1 | 5.19 |  | 46.9 | 39.0 | 47.3 | 54.5 |  | 46.1 | 5.99 |  | 32.7 | 54.3 | 164.6 | 379.6 |
| p-C | 54.7 | 63.2 | 50.5 | 47.3 |  | 53.9 | 5.52 |  | 45.3 | 36.8 | 49.5 | 52.7 |  | 22.3 | 27.4 |  | 27.4 | 44.6 | 150.0 | 319.1 |
|  |  |  |  |  |  |  |  |  |  |  |  |  |  |  |  |  |  |  |  |  |
| ID | 78.0 | 85.1 | 76.5 | 71.2 |  | 77.7 | 3.19 |  | 22.0 | 14.9 | 23.5 | 28.8 |  | 22.3 | 11.1 |  | 12.3 | 16.7 | 66.0 | 161.2 |
| SK | 83.4 | 90.3 | 84.8 | 82.2 |  | 85.2 | 1.83 |  | 16.6 | 9.7 | 15.2 | 17.8 |  | 14.8 | 10.5 |  | 8.23 | 9.60 | 37.6 | 88.5 |

^a^Percentage difference =(RF value of L-WS-RF value of G-WS/RF value of L-WS) × 100.

^b^Relative recovery = RF value of G-WS/RF value of L-WS × 100.

^c^Actual concentration (ppb) = Ideal concentration of G-WS × Relative recovery (%) / 100.

FSC (fixed standard concentration): analysis of 10, 20, 50, and 100 mL of the prepared G-WS at 45.1 ppb (FSC-1), 90.2 ppb (FSC-2), 225 ppb (FSC-3), and 451 ppb (FSC-4).

Theoretical concentration of gaseous working standard (G-WS). The second L-WS was vaporized at 280°C and swept into a 20 L polyester aluminum (PEA) bag

The mixing ratios were computed under the assumption that all liquid was converted into a gas phase standard without any loss.

Vaporization parameters: (1) nitrogen (>99.999%), (2) flow rate: 1000 mL min-1, (3) sweep time: 20 min, and sample volume: 20 L.

**Table S7:** Henry’s law constants (HLC, mol.kg-1.Pa-1) and sorbent capacities (Cap, mg.g-1, after 15 L loading)

for 14 VOCs on 3 MOFs and 3 commercial sorbents and VOC loss factors (LF) to system components

| Order | VOC | Tenax TA | | | Carbopack X | | | Carboxen 1000 | | | Eu-MOF | | | MOF-5 | | | MOF-199 | | |
| --- | --- | --- | --- | --- | --- | --- | --- | --- | --- | --- | --- | --- | --- | --- | --- | --- | --- | --- | --- |
|  |  | HLC | Cap | LF | HLC | Cap | LF | HLC | Cap | LF | HLC | Cap | LF | HLC | Cap | LF | HLC | Cap | LF |
| 1 | B | 0.33 | 0.25 | 0.055 | 0.55 | 0.42 | 0.06 | >8.3 | 4.8 | 0.065 | 1.2 | 1 | 0.05 | ---- | ---- | ----- | >2.1 | >1.1 | 0.05 |
| 2 | T | 0.32 | 0.24 | 0.055 | 0.86 | 0.62 | 0.075 | >12 | 5.4 | 0.075 | 1.2 | 0.95 | 0.065 | ---- | ---- | ----- | >5.3 | >2.6 | 0.05 |
| 3 | p-X | 0.38 | 0.27 | 0.057 | 1.9 | 1.5 | 0.08 | >17 | >6 | 0.1 | 1.1 | 0.76 | 0.08 | ---- | ---- | ----- | >11 | >5.2 | 0.05 |
| 4 | S | 0.3 | 0.2 | 0.095 | 1.7 | 1.2 | 0.12 | >19 | >6 | 0.1 | 1.3 | 0.85 | 0.1 | ---- | ---- | ----- | >12 | >4.9 | 0.1 |
| 5 | ACA | ---- | ---- | -0.47 | 0.46 | 0.2 | 0.21 | ----- | ---- | 0.5 | **3.6** | **0.83** | 0.55 | 1.3 | 0.51 | 0.36 | 3 | 0.89 | 0.51 |
| 6 | PPA | ---- | ---- | -0.48 | 0.59 | 0.33 | 0.24 | 12 | 4 | 0.5 | **3.6** | **1.1** | 0.55 | 1.5 | 0.78 | 0.35 | 5.7 | 2.1 | 0.55 |
| 7 | IBA | 0.56 | 0.2 | 0.35 | 1.6 | 1 | 0.16 | 7.8 | 3.4 | 0.37 | 2.8 | 1.4 | 0.35 | 1.1 | 0.74 | 0.18 | 5.4 | 2.4 | 0.48 |
| 8 | BTA | 1.2 | 0.65 | 0.1 | 3 | 2.3 | 0.055 | 11 | 4.5 | 0.25 | 5.1 | 3 | 0.16 | 1.4 | 1 | 0.07 | 6.4 | 3.1 | 0.4 |
| 9 | IVA | 2.2 | 1.3 | -0.04 | 4.3 | 3.2 | 0.033 | >15 | >5.5 | 0.15 | 6.6 | 3.9 | 0.11 | 2.4 | 1.9 | 0 | 8 | 4.1 | 0.32 |
| 10 | VLA | 2.9 | 1.7 | 0.13 | 4.9 | 3.2 | 0.011 | >15 | >6 | 0.15 | 11 | 5.1 | 0.11 | 3.3 | 2.4 | 0 | 15 | 6 | 0.25 |
| 11 | PhAl | 0.61 | 0.7 | 0.13 | 1.7 | 2.5 | 0.11 | >15 | >10 | 0.2 | 1.3 | 1.5 | 0.19 | ---- | ---- | ----- | 12 | 13 | 0.1 |
| 12 | p-C | 1.5 | 1.8 | 0.14 | 2.2 | 2.7 | 0.25 | >15 | >10 | 0.2 | 2.7 | 3 | 0.19 | 1.1 | 1.5 | 0.12 | 17 | 15 | 0.1 |
| 13 | ID | 1.6 | 1.1 | 0.25 | 2.5 | 1.8 | 0.35 | 1.7 | 0.7 | 0.62 | 2.7 | 1.5 | 0.37 | 1.8 | 1.4 | 0.17 | 10 | 4.5 | 0.55 |
| 14 | SK | 3.1 | 2.2 | 0.14 | 4.6 | 3.3 | 0.22 | 5 | 2.1 | 0.5 | 5.7 | 3 | 0.27 | 2.9 | 2.5 | 0.06 | 20 | 7.7 | 0.35 |

**Table S8.** DFT 0 K adsorption enthalpies and estimated 298 K Gibbs free energies (kJ.mol^-1^) for representative chemical species on two different MOFs examined in this study as compared to other sorbents.

| Order | Guest /host | Adsorption enthalpies (ΔH) Gibbs free energies (ΔG)^a^ (kJ.mol^-1^) | | | | Experimental ΔH  (kJ.mol^-1^) | | |
| --- | --- | --- | --- | --- | --- | --- | --- | --- |
|  | Molecules | MOF-5  ΔH | MOF-199  ΔH | MOF-5  ΔG | MOF-199  ΔG | Tenax TA | Carbotrap B | Graphitized carbon black |
| 1 | Toluene | -40.9 | -76.8 | -4.0 | -39.9 | -53.7^b^, -78.7^e^ | -31.7^c^ | -47.8^c^ |
| 2 | Butyric acid | -41.7 | -77.6 | -1.9 | -37.8 | -67.2^d^ | nd | nd |
| 3 | Phenol | -46.5 | -90.2 | -10.2 | -53.9 | -78.0^d^ | nd | nd |
| 4 | Indole | -47.8 | -94.7 | -9.9 | -56.8 | nd | nd | nd |

The reference order used in this table was, a) [8], b) [9], c) [10], d) [11], e) van’t Hoff plot of ln(BTV) vs. 1/T. BTV data from: http://www.sisweb.com/index/referenc/tenaxta.htm (accessed Oct. 2015)

**Table S9.** Preparation of vaporized gaseous samples of BTXS, VFAs, phenols, and indoles and their corresponding concentration levels used for the sorptive removal analysis.

| Order | Compounds | L-WS Conc. | Injection vol. | N_2_ flow rate | Sampling time | Total sampling | Ideal conc. | Ideal pp.^a^ | Measured conc | Sampling bag pp. | SVP^c^ at 25°C | Recovery |
| --- | --- | --- | --- | --- | --- | --- | --- | --- | --- | --- | --- | --- |
|  |  | used for vaporization (ng µL^-1^) | µL | mL min-1 | (min) | volume (L) | (ppb) | (mPa) | (ppb) | (mPa) | (mPa) | (%) |
| 1 | B^b^ | - | - |  |  |  | 100 | 10.0 | 98.3 | 9.83 | 12638961 | 98.3 |
| 2 | T^b^ | - | - |  |  |  | 100 | 10.0 | 83.3 | 8.33 | 3786355 | 83.3 |
| 3 | p-X^b^ | - | - |  |  |  | 100 | 10.0 | 76.5 | 7.65 | 1178570 | 76.5 |
| 4 | S^b^ | - | - |  |  |  | 100 | 10.0 | 72.1 | 7.21 | 853263 | 72.1 |
| 5 | ACA | 649 |  |  |  |  | 264 | 26.4 | 101 | 10.1 | 2097161 | 38.1 |
| 6 | PPA | 613 |  |  |  |  | 202 | 20.2 | 104 | 10.4 | 470628 | 51.5 |
| 7 | IBA | 600 |  |  |  |  | 167 | 16.7 | 89.9 | 8.99 | 241313 | 53.8 |
| 8 | BTA | 594 |  |  |  |  | 165 | 16.5 | 88.1 | 8.81 | 219982 | 53.4 |
| 9 | IVA | 575 |  |  |  |  | 138 | 13.8 | 73.4 | 7.34 | 58662 | 53.2 |
| 10 | VLA | 582 | 20 | 1000 | 20 | 20 | 139 | 13.9 | 69.7 | 6.97 | 26131 | 50.1 |
| 11 | PhAl | 1340 |  |  |  |  | 348 | 34.8 | 166 | 16.6 | 46663 | 47.7 |
| 12 | p-C | 1339 |  |  |  |  | 303 | 30.3 | 141 | 14.1 | 14665 | 46.6 |
| 13 | ID | 1343 |  |  |  |  | 280 | 28.0 | 92.5 | 9.25 | 1627 | 33.0 |
| 14 | SK | 1330 |  |  |  |  | 248 | 24.8 | 70.5 | 7.05 | 739 | 28.4 |

^a^pp : partial pressure; ^b^the samples was injected as a gaseous standard; ^c^the vapor pressure data was collected from Syracuse research corporation database (http://esc.syrres.com/fatepointer/search.asp)

**(a) MOF-5**

**(b) Eu-MOF**

**(c) MOF-199**

**Figure S1:** N_2_ adsorption isotherms of MOF-5, Eu-MOF, and MOF-199

(a) Carbopack X (CP-X)

**Figure S2**. Mass sorbed apportioned to mass sorbed on selected sorbent and total sorbed mass (sorbent + losses to system components and Henry's law constant (HLC) versus total mass loaded.

(b) Tenax TA (T-TA)

…

~Continued

(c) Eu-MOF

**Figure S3**. FTIR spectra for (a) MOF-5, (b) Eu-MOF, and (c) MOF-199 before and after the adsorption experiment.

~Continued

**References**

[1] Millward, A. R.; Yaghi, O. M. Metal-organic frameworks with exceptionally high capacity for storage of carbon dioxide at room temperature. *J. Am. Chem. Soc.* **2005**, *127*, 17998-17999.

[2] Kumar, P.;Paul, A.; Deep, A. Sensitive chemosensing of nitro group containing organophosphate pesticides with MOF-5. *Microporous Mesoporous Mater.* **2014**, *195*, 60-66.

[3] Choi, J. R.;Tachikawa, T.;Fujitsuka, M.; Majima, T. Europium-based metal−organic framework as a photocatalyst for the one-electron oxidation of organic compounds. *Langmuir* **2010**, *26*, 10437-10443.

[4] Heinke, L.;Gu, Z.; Wöll, C. The surface barrier phenomenon at the loading of metal-organic frameworks. *Nat. Commun.* **2014**, *5*.

[5] Xu, B.;Guo, H.;Wang, S.;Li, Y.;Zhang, H.; Liu, C. Solvothermal synthesis of luminescent Eu(BTC) (H_2_O) DMF hierarchical architectures. *Cryst. Eng. Comm.* **2012**, *14*, 2914-2919.

[6] Zhang, Q.;Yu, J.;Cai, J.;Song, R.;Cui, Y.;Yang, Y.;Chen, B.; Qian, G. A porous metal–organic framework with –COOH groups for highly efficient pollutant removal. *Chem. Commun.* **2014**, *50*, 14455-14458.

[7] Farha, O. K.;Eryazici, I.;Jeong, N. C.;Hauser, B. G.;Wilmer, C. E.;Sarjeant, A. A.;Snurr, R. Q.;Nguyen, S. T.;Yazaydın, A. Ö.; Hupp, J. T. Metal–organic framework materials with ultrahigh surface areas: Is the sky the limit? *J. Am. Chem. Soc.* **2012**, *134*, 15016-15021.

[8] Campbell, C. T.; Sellers, J. R. The entropies of adsorbed molecules. *J. Am. Chem. Soc.* **2012**, *134*, 18109-18115.

[9] Kroupa, A. n.;Dewulf, J.;Van Langenhove, H.; Vı́den, I. Breakthrough characteristics of volatile organic compounds in the −10 to +170 C temperature range on Tenax TA determined by microtrap technology. *J. Chromatogr. A* **2004**, *1038*, 215-223.

[10] Foley, P.;Gonzalez-Flesca, N.;Zdanevitch, I.; Corish, J. An investigation of the adsorption of C5-C12 hydrocarbons in the ppmv and ppbv ranges on Carbotrap B. *Environ. Sci. Technol.* **2001**, *35*, 1671-1679.

[11] Kalaschnikova, E.;Kiselev, A.; Shcherbakova, K. Gas chromatographic investigation of adsorption equilibria on graphitized thermal carbon black. *Chromatographia* **1974**, *7*, 22-25.
